# Exploring Ultrasound and Microwave-Assisted Accelerated Aging of Jerez Vinegar: Impacts on Phenolic, Volatile, Colorimetric, and Sensory Properties

**DOI:** 10.3390/foods14213665

**Published:** 2025-10-27

**Authors:** Reyhan Selin Uysal, Hanán Issa-Issa, Ángel A. Carbonell-Barrachina, Esther Sendra

**Affiliations:** 1 Department of Genetics and Bioengineering, Istanbul Bilgi University, Istanbul 34060, Turkey; 2 Grupo de Investigación “Calidad y Seguridad Alimentaria”, Centro de Investigación e Innovación Agroalimentaria y Agroambiental (CIAGRO-UMH), Universidad Miguel Hernández de Elche, Carretera de Beniel, km 3.2, 03312 Orihuela, Alicante, Spain; hissa@umh.es (H.I.-I.); angel.carbonell@umh.es (Á.A.C.-B.); esther.sendra@umh.es (E.S.)

**Keywords:** *Sherry* vinegar, ultrasonic field, microwave field, fast aging, LC–MS/MS, GC–MS, sensory profile, chromatic properties

## Abstract

Jerez vinegar is a high-quality wine vinegar produced in the Vinagre de Jerez denomination of origin (Spain) and is traditionally aged in wood barrels for over 10 years. Considering the long aging process, a practical technique to accelerate the aging process was simulated. This study aimed to evaluate ultrasound and microwave treatments as alternative aging methods for fresh Jerez vinegars with oak chips, and to investigate their effects on phenolic content, volatile compounds, and colorimetric and sensory properties. Fresh control samples with oak chips were treated using ultrasound (US) in an ultrasonic bath three times: 0.5 h (US1), 2 h (US2), and 10 h (US3). Microwave (MW) treatments were performed using a domestic microwave oven with three power/time combinations: 640 W for 10 min (MW1), 640 W for 20 min (MW2), and 800 W for 10 min (MW3). Compared with the fresh control (4230 µg/kg), US- and MW-treated samples showed a significant reduction in total phenolic content, decreasing to 3943 µg/kg in the US1 sample and to 3988 µg/kg in the MW2 treatment. Moreover, volatile substances significantly decreased from 1019 mg/L in the fresh control to 623 mg/L in the US3 treatment and 716 mg/L in the MW1 sample. Regarding sensory properties, US3 and MW1 treatments exhibited marked distinctions in certain odor and flavor attributes when compared with the fresh control. As a result, both techniques modified the phenolic, volatile and sensory profiles. Further research is needed to fully mimic the aging process, but US has proven to be a promising technique.

## 1. Introduction

Aged vinegars are an enological product highly valued by consumers, with various types distinguished by the raw products used and the production process. Jerez (Sherry) vinegar is one of the most renowned examples globally [1]. It is a premium and high-quality product produced in the region of Cadiz, Spain under the protection of the Vinagre de Jerez denomination of origin (DO). This geographical indication, recognized by Spain and Europe, highlights the product’s significance and heritage within its region of origin [2]. In addition to Vinagre de Jerez (JV), other aged wine vinegars produced and sold under a specific protected designation of origin (PDO) include “Vinagre de Condado de Huelva” and “Vinagre de Montilla-Moriles” [3]. JV, produced in geographically indicated regions, is made from Sherry wines obtained from several varieties of Palomino, Pedro Ximenez, and Muscatel grapes, using a traditional aging technic known as the “Soleras and Criaderas” system [4].

Jerez vinegars possess special sense features—bright color, intense aroma, and rich flavor—thanks to their unique production technique. This traditional method involves a dynamic aging system in which the vinegar undergoes oxidative aging in wooden barrels while simultaneously undergoing a slow acetification process. As both aging and acetification take place in oak barrels, significant changes in chemical, physical composition, and sensory characteristics occur throughout the process [5]. Barrel aging involves a slow oxygenation process, resulting in the polymerization and oxidation of aroma profiles [6], a desired aroma profile for the end product, the extraction of bioactive compounds from the wood barrels [7], and an enhanced flavor and taste of the product [8]. Based on the duration of aging, Jerez vinegars are classified as “Vinagre de Jerez (VJ)” when aged for at least 6 months up to 2 years, “Reserva” for a minimum of 2 to 10 years, and “Gran Reserva” for aging periods of 10 years or more [9]. As seen from the long process duration, this traditional method requires a large amount of space and a long maturation and aging period, which leads to reduced production efficiency. Although the traditional process is well-accepted by consumers due to its quality, it presents several challenges at the industrial scale. Barrels are not cost-efficient, require regular maintenance, consume significant time and space, and need to be replaced periodically [10]. Additionally, as barrels age, certain microorganisms may grow, leading to unpleasant odors or flavors, and potential hygiene issues.

In response to the current needs of the food industry regarding processing and preservation technologies, novel techniques are being incorporated to address these demands. To point out this issue, several types of research have been performed on various kinds of fermented wine-based products that undergo traditional long aging processes. In this manner, in recent years, ultrasound and microwave technologies have been proposed as physical methods to replace traditional long-duration methods in food technologies [11,12,13,14].

Ultrasound technology, particularly low-frequency high-energy power features, can be utilized in several fields of food process and preservation and to accelerate the process of vinegar aging. The effect of ultrasound may stem from the acoustic cavitation which generates the collapse of bubbles, leading to localized high temperature and pressure. This enhances chemical reactions such as oxidation, anthocyanin dimerization, and modifications in volatile and phenolic compounds, ultimately influencing the physicochemical and colorimetric characteristics of the samples [15]. These changes accelerate the aging process by promoting chemical reactions that control the physical, chromatic, and sensory properties of vinegar [16,17]. In a previous study, ultrasound was applied to accelerate the extraction of phenolic compounds from wood into wine spirits, simulating the process happening during wine aging [18]. Additionally, in the winemaking process, the application of ultrasound technology has been promoted in the processes of maceration [19], fermentation, and aging [20]. The acceleration of aging in vinegar production has also been studied in various types of vinegar, including balsamic vinegar [21], crabapple vinegar [22], Zhenjiang vinegar [23], and grape pomace vinegar [24].

On the other hand, microwave technology has recently been highlighted for its ability to induce chemical reactions that accelerate the aging of wine and vinegar [25]. Microwave technology exhibits non-ionizing electromagnetic waves with frequencies between 0.3 and 300 Ghz, frequencies which lead to molecular movement by migration of ions and the rotation of molecules with permanent dipoles, especially in liquids inducing thermal, electric field, and other nonthermal effects [26]. Similarly, microwave treatment rapidly increases molecular motion and thermal energy, which can accelerate reactions such as esterification and oxidation, thereby reducing processing time. In one study, microwaves were used to shorten the lees aging process in red wines, effecting the volatile components by the acceleration of yeast autolysis [27]. Olfactometric and sensory evaluations of red wines were conducted following accelerated aging using microwave or ultrasound treatments [28]. Although the primary effects of these treatments are chemical, they may also influence microbial activity during acetification by altering the local environment. However, microbial inactivation was not the focus of the present study as the treatments were applied solely to accelerate the aging process.

According to the literature reviewed, there is a growing interest in emerging technologies aimed at accelerating the aging process of vinegar and oenological products. To the best of our knowledge, no previous study has investigated the use of ultrasound or microwave technologies in combination with wood chips to accelerate the aging of fresh Jerez vinegars—typically aged for at least six months (the type of Vinagre de Jerez). As mentioned, these treatments can accelerate chemical reactions and partially mimic the extraction processes that occur during traditional aging in oak barrels, affecting aroma, phenolic compounds, colorimetric properties, and sensory profiles. Furthermore, no comparative analysis of these two methods regarding their effects has been reported. This study thus provides a promising assessment of the potential of these techniques to partially substitute traditional aging methods.

Consequently, this study aimed to evaluate the effects of ultrasound and microwave technologies on accelerating vinegar aging and to assess how different treatment parameters influence phenolic compounds, volatile components, color, and sensory properties. Additionally, we sought to identify the most effective technology for accelerating reactions and enhancing extraction, and to propose a rapid, innovative method applicable to barrel-aged vinegars in the food and beverage sector.

## 2. Materials and Methods

### 2.1. Materials and Chemicals

Chemicals used in the study are as follows: Methanol, acetonitrile (all LC–MS grade, ≥99.9%), sodium chloride and 1-hexanol (all reagent grade, 98%) for LC–MS/MS analysis were purchased from Panreac Applichem (Darmstadt, Germany). Formic acid (LC–MS grade, ≥99.9%) was purchased from VWR Chemicals BDH (Leuven, Belgium). The anthocyanins analyzed in this study are as follows: Cyanidin-3-*O*-glucoside, delphinidin-3-*O*-glucoside, malvidin-3-*O*-glucoside, pelargonidin-3-*O*-glucoside, peonidin-3-*O*-glucoside, petunidin-3-*O*-glucoside, cyanidin-3,5-di-*O*-glucoside, delphinidin-3,5-di-*O*-glucoside, malvidin-3,5-di-*O*-glucoside, pelargonidin-3,5-di-*O*-glucoside, peonidin-3,5-di-*O*-glucoside; malvidin-3-*O*-galactoside, cyanidin-3-*O*-rutinoside, cyanidin-3-*O*-arabinoside, and pelargonidin-3-*O*-rutinoside; flavonols: myricetin-3-*O*-glucoside, quercetin-3-*O*-glucoside, quercetin-3-*O*-galactoside, quercetin-3-*O*-rutinoside, quercetin-3-*O*-glucuronide, and quercetin-3-*O*-glucopyranoside; flavones: luteolin-7-*O*-glucoside; phenolic acids: caffeic acid, neochlorogenic acid, chlorogenic acid, and gallic acid; flavanones: eriotricin, hesperidin, cynarine, and rutin; and stilbenes: (*E*)-resveratrol were supplied from Cymit Química (Barcelona, Spain).

### 2.2. Vinegar Samples

Samples of fresh control (non-aged) and Vinagre de Jerez (Crianza) traditional Sherry wine vinegar samples that had been aged for at least 6 months were kindly provided by El Majuelo, a producer of traditional Jerez vinegar from the Jerez de la Frontera region of Cádiz, Spain. All of the samples were analyzed in duplicate (from two batches of the same kind of vinegar). The Jerez vinegar was produced from Sherry wine prepared with Palomino grapes. All traditional Jerez vinegar varieties must be made exclusively from Sherry wines, following the regulations of the protected designation of origin (PDO) and the European classification system, with specific aging periods established by the PDO. The aging process takes place in American oak casks applying the traditional “Criadera y Solera” method and the Crianza sample in the study was aged using this technique.

### 2.3. Accelerated Aging by Ultrasound and Microwave Techniques

Fresh vinegar samples were aged using non-traditional methods, namely ultrasound (US) and microwave (MW) techniques. To mimic the traditional aging process in American oak barrels, medium-toasted American oak chips supplied from a local barrel supplier (Alcofermbrew, Olsztyn, Poland) were used in both aging processes. The chip size was 5–15 mm in length, 5–10 mm in width, and 2–3 mm in thickness. The chips were kept in boiling water for 15 min to precondition them before being used in the treatments. The preconditioning setup of chips is shown in Figure S1. The ultrasound and microwave treatments were performed on a 250 mL sample with a percentage of 1% (*w*/*v*) oak chips in 500 mL borosilicate flask glass bottles (autoclavable, with screw caps). These bottles provided sufficient headspace (~250 mL) to allow effective acoustic energy transmission while minimizing evaporation during treatments.

The accelerated aging of fresh vinegar with 1% (*w*/*v*) of oak chips was carried out using an ultrasonic bath (Ultrasons-HD, 3000864, J.P. Selecta S.A., Barcelona, Spain) operating at a frequency of 50 kHz and a power of 800 W. Ultrasound treatments were applied to glass flask bottles with 250 mL of fresh sample in three periods: 0.5 h (US1), 2 h (US2), and 10 h (US3), representing short, medium, and long sonication periods, respectively [11]. The longest treatment (10 h) was performed with on/off pulses, consisting of 90 min of continuous sonication followed by a 2 min pause to prevent excessive temperature rise. During each 2 min pause, the bath was a static system in which heated water was partially drained, and the temperature was maintained below 35 °C by adding ice (produced from an ice machine) whenever it approached 30 °C. The bath temperature was continuously monitored using the built-in display of the ultrasonic bath and verified at intervals with a manual thermometer immersed in the water. To maintain a constant level, the water level in the ultrasound bath was kept at 2 cm above the vinegar level in the bottles by replenishing it with the same amount of fresh water and ice as described above. The ice machine, ultrasonic bath, and system setup are shown in Figure S2.

As a second technique, microwave treatments were applied to glass flask bottles containing 250 mL of fresh vinegar with 1% (*w*/*v*) oak chips. A domestic LG household microwave oven (model MH6339H, LG Electronics Inc., Nanjing, China) with an input power of 2250 W, a maximum output power of 800 W and a frequency of 2450 MHz was used. Three different microwave power and time combinations were applied: MW1 (640 W for 10 min), MW2 (640 W for 20 min), and MW3 (800 W for 10 min). During the treatments, sample temperatures were monitored using an infrared (IR) camera (FLIR E6-XT, FLIR Systems AB, Täby, Sweden), with on/off pulses every 2 min to maintain the temperature below 40 °C. During each 2 min “on” pulse, the temperature increased by approximately 3 °C for MW1 and MW2 (640 W) and by approximately 5 °C for MW3 (800 W). The images of the bottles during all MW treatments were taken by IR camera and are presented in Figures S3–S5.

### 2.4. Analysis of Phenolics by Liquid Chromatography–Mass Spectrometry (LC–MS/MS)

Fresh control and all treated vinegar samples were diluted (1:1, *w*/*v*) by the addition of an extractant made of methanol/water/formic acid (80:19.9:0.1, *v*/*v*). Then, diluted vinegar samples were filtered via a 0.45 µm pore size membrane filter before injection into the LC–MS/MS system. Phenolic components, in particular anthocyanins and polyphenols, were analyzed using a Shimadzu LC–MS/MS 8050 triple quadrupole mass spectrometer (Shimadzu Corporation, Kyoto, Japan) equipped with an electrospray ionization (ESI) as a source operating in negative and positive modes. The LC–MS/MS analysis was conducted following the procedure implemented in the study by Uysal, et al. [7]. The mobile phase for anthocyanins and polyphenols is made of two solvents: (i) solvent A, water/formic acid (99.9:0.1, *v*/*v*) and (ii) solvent B, acetonitrile/formic acid (99.9:0.1, *v*/*v*). Anthocyanins were eluted under the following conditions: 0.4 mL min^−1^ flow rate and 50 °C, isocratic conditions for 2 min with 95% A, from 2 to 10 min linear gradient of 5% to 95% acetonitrile with 0.1% formic acid (B), isocratic conditions with solvent B continued for 10–11 min, then returned to initial conditions of 95% A in 1 min, and isocratic conditions with 95% of 1% aqueous formic acid for 4 min followed by washing and reconditioning the column. Moreover, the elution conditions of polyphenol compounds were as follows: 0.4 mL min^−1^ flow rate and 40 °C, isocratic conditions for 2 min with 90% A, from 2 to 17 min linear gradient of 10% to 100% acetonitrile with 0.1% formic acid (B), then returning to initial conditions of 90% A in 4 min, followed by washing and reconditioning the column. The injection volume of the samples was 10 µL for both anthocyanin and polyphenol compounds. Two MS experiments were conducted: one for anthocyanins in positive mode and one for polyphenols in negative mode, both before and after fragmentation. Characterization of the single components was based on their retention times and accurate molecular masses. The MS conditions were as follows: Analyses were carried out with full scan mode, and data-dependent MS scanning from *m*/*z* 100 to 1800, via collision-induced fragmentation experiments conducted using argon as the collision gas. The capillary voltage was 4.0 kV. The capillary temperature was set at 300 °C and the source heater temperature was set at 250 °C, while desolvation gas flow rate was 160 L/h. An analysis of anthocyanin and polyphenols was carried out in duplicate as technical replicates for each sample.

All statistical parameters related to the quantitative determination of phenolic compounds (flavonols, anthocyanins, flavanones, phenolic acids, stilbenes, and flavones) are presented in Table S1. These include the calibration model equations, determination coefficients (*R*^2^), relative standard deviations (RSD), and limits of detection (LOD) for each phenolic compound analyzed. The high *R*^2^ values and low RSD values confirm the reliability and repeatability of the analytical method, while the obtained LOD values indicate sufficient sensitivity for accurate quantification within the tested concentration range.

### 2.5. Analysis of Volatile Compounds by Gas Chromatography–Mass Spectrometry (GC–MS)

The volatile compounds in the vinegar samples were analyzed using a gas chromatography (GC-2030)–mass spectrometer (GCMS-TQ8040 NX, Shimadzu Corporation, Kyoto, Japan) combined with the headspace solid-phase microextraction (HS-SPME) technique. The analysis followed a procedure outlined in a previous study [6]. Prior to analysis, the samples were prepared as follows: A 10 mL aliquot of the sample was transferred into a 20 mL vial, and 100 μL of 1-hexanol was added as an internal standard. To saturate the sample, 1 g of sodium chloride was incorporated. The vial was then securely sealed with a polypropylene cap featuring a PTFE/silicone septum. Extraction was carried out at a constant temperature of 40 °C for 40 min with agitation at 250 rpm using an AOC-6000 Plus autosampler (Shimadzu Corporation, Kyoto, Japan) equipped with SPME capability. The separation and identification of analytes were performed using both InertCap Pure-WAX (30 m × 0.25 mm × 0.25 µm) capillary columns (Teknokroma, Barcelona, Spain). After extraction, volatile analytes were desorbed from the DVB/CAR/PDMS fiber coating in the GC injection port at 210 °C for the Pure-WAX column for 1 min in a 1:10 split mode. The temperature program for both columns was set as follows: an initial temperature of 50 °C, held for 1 min, then increased to 100 °C at a rate of 2 °C/min, followed by a rise to 180 °C at 3 °C/min, and finally increased from 180 °C to 230 °C at a rate of 20 °C/min, where it was held for 5 min. Helium was used as the carrier gas at a flow rate of 0.6 mL/min. The MS conditions for the Pure-WAX column were as follows: capillary direct interface temperature of 240 °C, ion source temperature of 210 °C, positive ionization mode, and an *m/z* scan range of 40–400 amu.

Compound identification was conducted based on the retention index, comparison of EI mass spectra, and a C6–C40 alkane mixture (Sigma-Aldrich, Steinheim, Germany). Each volatile compound was quantified using its relative area compared with the internal standard (1-hexanol, 1000 mg/L). The relative concentration of an analyte was calculated using the following formula: relative concentration = (peak area of the compound × concentration of the internal standard)/peak area of the internal standard. Each sample was analyzed in triplicate as technical replicates.

### 2.6. Analyses of Colorimetric Characteristics by UV–Vis Spectrophotometer 

The colorimetric properties of vinegar samples, including (i) tonality, (ii) color intensity, and (iii) color density, were analyzed following the procedure outlined in [29]. Absorbance measurements were conducted using a UV–visible spectrophotometer (UV-1280, Shimadzu Corporation, Kyoto, Japan). The colorimetric components of the vinegar samples were also assessed at wavelengths of 420 nm (yellow components), 520 nm (red components), and 620 nm (blue components). Based on these measurements, color intensity (IC), tonality (T), and color density (D) were determined using the following formulas:

IC = A420 + A520 + A620

T = A420/A520

D = A420 + A520

The Glories color index percentages for yellow (Y%), red (R%), and blue (B%) in the vinegar samples were calculated based on absorbance data recorded at 420, 520, and 620 nm [30]. Colorimetric measurements were conducted in triplicate.

### 2.7. Descriptive Sensory Analysis

A panel of seven evaluators, consisting of three females and four males aged between 30 and 55 years, assessed the vinegar samples at the Food Quality and Safety (CSA) research group facilities of Universidad Miguel Hernández de Elche (UMH). The descriptors and questionnaire were developed based on guidelines from the Foundation of Organization of Evaluation Conformity and Food Certification (OECCA, Cádiz, Spain) [31] and on a methodology described in a study by Tesfaye, et al. [2]. For odor analysis (detection of volatile compounds outside the mouth), 20 mL of vinegar diluted threefold with water was served in non-transparent standard wine-tasting cups. For flavor analysis, encompassing odor, aroma (volatile compounds perceived in the mouth), basic tastes, and chemical feeling factors—as well as global attributes, 20 mL of vinegar diluted fivefold with water was provided. Appearance evaluation was conducted using transparent cups. Figure S6 shows the set-up: the first row of samples corresponds to appearance evaluation, the second row to odor analysis in covered non-transparent cups, and the final row to flavor evaluation samples. The sensory analysis was performed under controlled room conditions (18–20 °C) and illuminated with white light. Each sample was assigned a three-digit code and presented individually in a randomized order, with a 5 min interval between samples. Panelists were provided with water and unsalted crackers for palate cleansing between tastings. The sensory evaluation included the following attributes:

- Odor: overall odor, vinegar ID, winy character, raisin, ethyl acetate (chemical), alcohol/liquor, woody, fruity, spicy, vanilla, clove, toasted, nuts, and leather/old.
- Flavor: overall flavor, vinegar ID, winy character, raisin, ethyl acetate (chemical), alcohol/liquor, woody, fruity, vanilla, clove, toasted, nuts, and leather/old.
- Basic tastes: sweetness, sourness, and bitterness.
- Chemical sensations: astringency and pungency.
- Global attributes: aftertaste.
- Appearance: color and *untuoso* (texture).
- Defects: dirty (*sucio*), bacterial, cheese-like, and sawdust (wood shavings).

Panelists rated each attribute on a 0 to 10 scale, where 10 indicated extremely high intensity and 0 represented an extremely low or undetectable intensity. The present study was approved by the ethical committee of Universidad Miguel Hernández de Elche (PRL.DTA.ESN.13.21), and all trained panelists voluntarily consented to participate in the study.

### 2.8. Statistical Analysis

All experiments were conducted in triplicate, and the results expressed as mean. Statistical analyses were performed using XLSTAT Premium 2016 (Addinsoft, New York, NY, USA). A one-way analysis of variance (ANOVA) was used to determine significant differences in colorimetric characteristics and phenolics among the vinegar samples. Tukey’s multiple range test was applied as a post-hoc analysis, with statistical significance set at a *p*-value of less than 0.05.

Principal component analysis (PCA) was performed to evaluate the differences among samples based on their chemical composition and sensory profiles. Two PCA analyses were conducted for volatile compounds, phenolic compounds, and sensory attributes to visualize clustering patterns and identify key variables contributing to sample differentiation. The analysis aimed to explore the relationships between chemical composition and sensory perception, as well as to determine the impact of ultrasound and microwave treatments compared with the fresh control and traditionally aged (*Jerez*) samples. All PCA analyses were carried out using the XLSTAT Premium 2016 software (Addinsoft, New York, NY, USA), and the results expressed in terms of eigenvalues, variance explained by each principal component, and the contribution of individual variables.

## 3. Results and Discussion

### 3.1. Phenolic Components by LC–MS/MS

The total phenolic compounds—including anthocyanins, stilbenes, flavones, flavanones, flavonols, and phenolic acids—were detected by using the mode of ESI with LC–MS/MS. The content of all phenolic compounds analyzed in fresh control, Jerez, and treated vinegars (US1, US2, US3, and MW1, MW2, MW3) is presented in Table 1. In terms of total phenolic content, the highest value was found in the fresh control sample (4229 µg/kg), while the lowest was observed in the traditionally aged Jerez vinegar (2514 µg/kg). Among the treated samples, US1 (0.5 h) and MW2 (640 W for 20 min) resulted in 3942 µg/kg and 3988 µg/kg, respectively. These results indicate that all treatments caused a significant decrease in phenolic content compared with the fresh control sample. US1 and MW2 were identified as the most effective treatments in terms of phenolic reduction. Nevertheless, the lowest phenolic content overall was still found in the traditionally aged Jerez sample (aged for at least 6 months). It can be concluded that both ultrasound and microwave treatments, depending on the applied parameters, may influence the degradation or reduction of phenolic compounds.

The major phenolic family of grape source is anthocyanins, as a total of 15 derivatives of them were analyzed by LC–MS/MS, but only six types of these compounds, which are peonidin-3,5-di-*O*-glucoside, cyanidin-3-*O-*rutinoside, pelargonidin-3-*O-*glucoside, peonidin-3-*O*-glucoside, malvidin-3-*O*-glucoside, and malvidin-3-*O*-galactoside, could be determined with a trace level in some of the samples. Peonidin-3,5-di-O-glucoside was detected in the MW2 sample at a concentration of 11.9 µg/kg, while pelargonidin-3-*O*-glucoside was found in the Jerez sample at 15.3 µg/kg. The absence of other anthocyanins, aside from the effects of the applied treatments, may be attributed to their degradation during the acetification process in the conversion of wine to vinegar. Moreover, the vinification process also induced several reactions in mono-anthocyanins and led to polymerization with other molecules. Due to their instability, anthocyanins cannot be detected in fermented and aged vinegar types [7,32]. Additionally, the acetification process of Sherry wine to Jerez vinegar product [33] found a decrease (~50%) in the amount of free anthocyanins. This clarifies why vinegar products contain a lower number of anthocyanins than wine products.

The second important compound responsible for the color properties of grapes is *t*-resveratrol. The effect of ultrasound or microwave treatment on aging may not be related to changes in the amount of trans-resveratrol, as it was not detected in the fresh control sample. Like anthocyanins, the acetification process of red-wine vinegar led to a loss of *t*-resveratrol in wood [33,34]. One of the most significant impacts was observed in the flavanone group, specifically in eriotricin compounds. Eriocitrin was measured at 65.8 µg/kg in the fresh control sample and decreased to 24.8 µg/kg in US2 and 25 µg/kg in MW3. While hesperidin was present at trace levels in the fresh control sample, it was not detected in any of the aged samples. Furthermore, from the flavonol group, a significant decline was obtained in the quercetin-3-*O*-glucopyranoside and quercetin 3-*O*-glucoside from 202 to 161 µg/kg and 232 to 175 µg/kg, respectively, in US3 treatment. Quercetin-3-*O*-glucuronide declined from 1806 to 1738 µg/kg. Among these compounds, ultrasound treatment appears to have a greater impact on accelerating the aging process of Jerez vinegars. A study conducted on *Sherry* vinegars aged with ultrasonication and micro-oxygenation with different oak chips reported significant differences in phenolics, depending on the oak chip variety used [35].

Chlorogenic acid, among the phenolic acid derivatives, showed a significant decrease in the content of the US3 and MW1 samples, from 23.4 µg/kg to ND and to 14.8 µg/kg, respectively. Caffeic acid showed a significant decrease from 307 to 238 µg/kg in US2 and to 198 µg/kg in the MW1 sample. Lastly, gallic acid from phenolic acids was detected, showing a decline in content from 1191 µg/kg (fresh control) to 1120 (US1) and 1109 (MW3). The loss in these compounds might be related to the oxidation mechanism during the aging period [9]. This was also explained by the way in which an increase in aging time with the contact of oak chips decreased the level of phenolic acids [36]. 

Although the effect of accelerating aging has not been seen on all compounds, it is observed that both treatments have a significant effect on total phenolic components, some of them quite specifically. In another study, although the results of different types of woods investigated by accelerating *Sherry* vinegars via ultrasonic treatment comply with this study, a decrease in the phenolic content was detected [35]. As a result of this analysis, the impact of accelerating aging on phenolic compounds through a series of chemical interactions occurs between free and polymerized forms of anthocyanins, flavonol, and phenolic acids. As can be seen from the table, although a major decrease in the phenolics occurred in the traditional aged sample (*Jerez*), ultrasound shows little difference from the microwave technique in the effect of accelerating the aging process in terms of total phenolic compounds.

### 3.2. Volatile Compounds by GC–MS/MS

**Figure 1:**
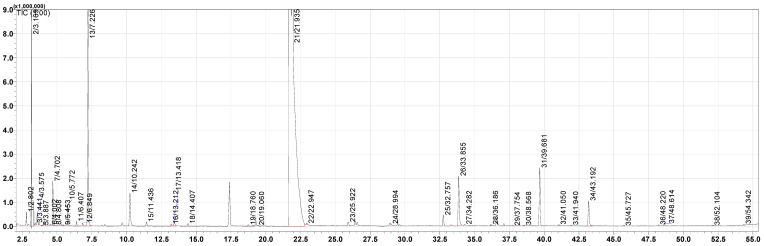
Model GC–MS chromatogram of volatile compounds identified in the fresh control sample.

Volatile compounds of all fresh and aged vinegar samples were analyzed using GC–MS/MS by applying the HS-SPME technique. A total of 39 volatile compounds were detected by the DB-Wax column, and the results are depicted in Table 2. A representative model chromatogram obtained from GC–MS/MS analysis, illustrating the separation of volatile compounds in the fresh control sample, is presented in Figure 1. Analyzed volatile compounds are divided into six chemical types depending on the presence of intensity: esters, acids, alcohols, aldehydes, ketones, phenolic compounds, and others (not in any specific family).

The main contributors to the volatile compounds sourcing from esters and organic acids constituted in the samples show a notable change depending on the alternative ageing treatments. Esters represent the highest diversity compounds among the volatile compound families, with 20 substituents, and they are responsible for floral, fruity, and nutty aromas. Ethyl acetate, isobutyl acetate, 2-methylbutyl acetate, and phenethyl acetate were found to be the most abundant substances in the fresh control sample with the amounts of 43.9, 12.3, 125, and 27.5 mg/L, respectively. Esters are mostly formed during the fermentation process of vinegars through the reaction between ethanol and organic acids [37,38]. Ethyl acetate, as one of the main ester components, may be released by the polymerization of ethanol and acetic acid with the loss of water and decreased to the content of 25.9 mg/L and 29.5 mg/L in aged with the treatment of US3 and MW1, respectively [8]. In comparison, the Jerez vinegar sample showed that the ethyl acetate content was measured at 32.5 mg/L, indicating that the alternative treatments were as effective as the traditional aging method. Although Jerez vinegar showed a higher ethyl acetate content, the lower levels observed in US3 and MW1 reflect the natural trend of decreasing ethyl acetate during aging, further supporting the ability of these treatments to mimic aging-related changes. Exhibition of a decline profile for ethyl acetate may be explained by volatilization or might be due to the change in esterification balance [39]. The second most abundant ester component, 2-methylbutyl acetate, was determined with a concentration of 125 mg/L and 36.6 mg/L in fresh control and Jerez, respectively. As a similar trend with ethyl acetate, the concentration has decreased to 61.6 mg/L (US3) and 58.2 mg/L (MW1) in the aged samples. Methyl acetate is produced during the vinegar process however and, like ethyl acetate [40], has shown a decline resulting from a possible loss of volatilization. Another ester component, phenethyl acetate, was also quantified at 27.5 mg/L in the fresh control sample, and at 15.3 mg/L and 18.1 mg/L in the US3- and MW1-treated samples, respectively. As the last one, the fourth abundant component isobutyl acetated presented a decrease, with the amounts of 12.3, 6.36, and 6.32 mg/L in fresh control, US3, and MW1 samples. As can be seen from the values, all of the ester components exhibit a general decline in the aged samples and both ultrasound and microwave treatments influence the aging process.

Secondly, as the most abundant chemical family in terms of quantity are organic acids with six components. The most abundant component was detected in acids and all chemical substances for acetic acid with concentrations of 716, 451, and 533 mg/L in the fresh control, US3 and MW1 samples, respectively. For the Jerez sample, acetic acid obtained a concentration of 314 mg/L. As evident from the results, ultrasound and microwave treatments have an influence comparable to the traditional system on the condensation reactions involving acetic acid during the aging process. Acetic acid, which is responsible for the main flavor profile of vinegar produced during the acetification process, may show a distinct decline in content due to evaporation and condensation reactions with ethanol [41]. Moreover, a similar decrease in the concentration of acetic acid was observed during the aging of traditional balsamic vinegar in oak barrels [42]. The second main component is isovaleric acid with a content of 23.1 mg/L and 25.4 mg/L in the samples of fresh control and Jerez, respectively. A quantity of 9.57 mg/L was quantified in Gran Reserva. Due to this observed decrease, it is believed that isovaleric acid is involved in the esterification reactions of short-chain carboxylic esters during the aging process [43]. As can be seen from the table, during aging time, it can be implied that all of these factors, including vaporization, esterification, and condensation reactions, play a significant role in the decrease in the content of acids.

The other chemical family found among the volatile compounds, that of the alcohols, differs from other components and has shown an increasing trend depending on the compound, especially during long aging processes of over ten years [6]. In this study, ethyl alcohol, one of the most abundant alcohol compounds, was found at concentrations of 2.91 mg/L, 3.04 mg/L, and 4.42 mg/L in the fresh control, US1, and MW3 samples, respectively. The slight increase in the amount of ethyl alcohol may have resulted from the hydrolysis of ethyl acetate into ethanol. Another alcohol compound, active amyl alcohol, was measured at 15.5 mg/L, 11.2 mg/L, and 13.5 mg/L in the fresh control, US2, and MW3 samples, respectively, with the highest level observed in the fresh control sample. The last compound, phenylethyl alcohol, showed a decrease in concentration from 12.7 mg/L in fresh control to 7.67 mg/L in US3 and 8.35 mg/L in MW1. A similar trend was observed in a study on Shanxi aged vinegars, where phenylethyl alcohol levels declined over time [44]. Hereby, although alcohols are initially produced during alcoholic fermentation, subsequent fermentation and aging processes can affect their levels differently, either increasing or decreasing concentrations due to evaporation (leading to a decline) or complex chemical reactions (resulting in an increase) occurring during aging.

Among aldehydes, isovaleraldehyde, furfural, and benzaldehyde were detected and identified in the samples. These compounds are specifically responsible for herbal, sweet, and floral odors [45]. Although they were present only in trace amounts, the impact of aging treatments was still observable. In the US and MW treatments, furfural concentration showed a slight increase from 0.56 mg/L to 0.83 mg/L (US1) and 0.78 mg/L (MW1). Typically, aldehydes exhibit a decrease during the early stages of aging, followed by a gradual increase over time [6]. The barrel-derived aromatic compound furfural may have been extracted from the wood chips used during the treatments. The second aromatic compound, benzaldehyde, showed a decrease from 2.52 mg/L in the fresh control to 1.17 mg/L in US3 and 1.40 mg/L in MW2. A similar decline in benzaldehyde content was observed in the Jerez sample, where it was found at 0.23 mg/L. This reduction may be related to oxidation reactions [46]. Benzaldehyde is also recognized as one of the main aromatic aldehydes found in various vinegars, including Chinese traditional bran vinegar (Cupei), Italian balsamic vinegar, and Shanxi aged vinegar [45,47,48].

Furthermore, two ketone compounds were detected in the vinegar samples. Acetoin was the most abundant, with a concentration of 3.54 mg/L in the fresh control sample. 2-Nonanone was the second ketone identified, and its level decreased from 0.46 mg/L to 0.16 mg/L (US3) and 0.13 mg/L (MW1) during aging treatments. The aging processes using US and MW methods also led to a reduction in acetoin content, to 2.01 mg/L (US3) and 2.36 mg/L (MW1), respectively. A similar decrease was observed in the traditionally aged sample, where acetoin was found at 2.56 mg/L. Other components identified in low amounts in the samples included 2-ethylphenol (a phenolic compound) and linalool 3,7-oxide. Both compounds showed a decline in concentration in the alternatively aged and traditionally aged samples.

As shown in Table 2, the total volatile compound content significantly decreased from 1019 mg/L in the fresh control sample. As mentioned above, this decline is indicative of various processes, such as oxidation, evaporation, wood extraction, and esterification, playing a significant role during this period, as proved in the previous study [6]. The greatest similarity to the traditionally aged Jerez sample (449 mg/L) was observed in the US3 and MW1 treated samples, with volatile contents of 623 mg/L and 716 mg/L, respectively. These findings suggest that ultrasonic field and alternating magnetic field treatments effectively accelerated the aging of vinegar. These findings are consistent with the results obtained from previous studies on vinegar aged using ultrasound and magnetic field assistance, in comparison with fresh vinegar [49]. As observed by specific chemical families, the ultrasound treatment produced volatile compound profiles most similar to those of the traditionally aged Jerez sample (aged for at least 6 months). In particular, the third ultrasound treatment (10 h) showed the closest match to the Jerez sample in terms of volatile content. Therefore, ultrasound and microwave treatments can be proposed as novel accelerated aging methods, capable of delivering comparable quality in terms of flavor and aroma.

### 3.3. Colorimetric Characteristics

To determine the colorimetric properties of vinegar samples, UV–Vis measurements were taken at 420, 520, and 620 nm. Based on these measurements, color intensity (CI), tonality (T), color density (D), and the percentages of yellow, red, and blue were calculated. The values for all colorimetric characteristics of the samples are depicted in Table 3. The colorimetric properties particularly depend on phenolic compounds, as the color pigments originate from grapes. The fresh control sample showed the highest yellow pigment content (72.3%) but low levels of red (21.4%) and blue pigments (6.3%), when compared with other samples. On the contrary, the *Jerez* sample exhibited the lowest yellow pigment content (65.5%) and the highest levels of red (25.1%) and blue pigments (9.4%). The increase in red components during aging may have proportionally exceeded that of yellow components that led to this observed difference. The colorimetric properties of the aged samples with US and MW have not been changed significantly. The closest values of color properties to Jerez samples, obtained for ultrasound-treated samples with the lowest yellow component were obtained in US3 samples with 69.7% and the highest red (21.1%) and blue (9.2%) components. The MW-treated samples did not exhibit significant differences in color properties when compared with the fresh control. Although the total phenolic compounds showed a decline in the MW2-treated sample, this change did not significantly affect the color properties. Moreover, the total phenolic content was found to be lowest in the US-treated samples when compared with the MW-treated ones. This may explain why the US-treated sample exhibited the most pronounced differences in colorimetric characteristics.

The change in the color from yellow to red absorbance measurements might be explained with Maillard and caramelization reactions and finalized with browning agents [50,51]. In a study on Sherry vinegar, absorbance measurements were taken at 470 nm to assess the impact of aging time. A simultaneous increase in absorbance values was observed, which was attributed to the oxidation of polyphenolic compounds during the aging process [1]. In addition, while yellow pigment decreases, an increase in blue pigment was observed in the US3 and Jerez samples, with a remarkably close percentage of 9.2% and 9.4% for US3 and Jerez, respectively. The change in absorbance values at 420 nm, 520 nm, and 620 nm from fresh control to US3 and Jerez indicated an overall increase in respective CI values, from 1.426 to 1.82 and 3.127, and also respective D values, from 1.336 to 1.65 and 2.833. Conversely, there was a decline in the T values of the fresh control, US2, and Jerez samples, decreasing from 3.386 in the fresh control to 3.216 in US2 and 2.616 in Jerez. Regarding the tonality property, the US2 sample was found to be the closest to the Jerez sample, due to changes in the red-to-yellow ratio. The decrease in the T value may be attributed to the rise in red pigment and, indirectly, absorbance at 520 nm. The color properties of vinegars made up of red wine originate from the grape surface and wine. As known, grapes are rich in anthocyanins, tannins, and resveratrol compounds, which contribute to their unique color. As mentioned in previous chapters, it can be stated that the changes in the aging process, from either traditional or ultrasound treatment, facilitates copolymerization reactions between tannins and anthocyanin derivatives, resulting in the loss of these monomeric compounds and color pigments [7].

As a result, ultrasound treatment had a greater impact on the colorimetric properties of the fresh control sample when compared with the microwave technique. In particular, the sample treated with ultrasound for 10 h was found to be the most similar to the Jerez sample. This is consistent with the phenolic compound results as well. This result can be explained by the action of ultrasound, which causes the expansion of solvent molecules due to their elastic properties and creates cavities that collapse, causing localized increases in pressure and temperature. These pressures alter the plant tissues, thereby enhancing the extraction of compounds from the tissue matrix. It has also been explained that ultrasound can produce free radicals during the sonolysis of the solvent [52], which may lead to the formation of other oxidizing species. These species can participate in oxidation-based processes, such as the aging of most wine-derived products that come into contact with wood [35].

### 3.4. Sensory Properties

Analyzing the chemical changes that occur during the accelerated and traditional aging processes of vinegars and oenological products and evaluating their impact on sensory properties are particularly important for understanding the effects of acetification and oxidation. Therefore, all samples were analyzed in terms of sensory properties to distinguish the effect of alternative techniques compared with traditional ones. The sensory properties of vinegars are influenced by volatile compounds originating from raw materials and the aging process. Therefore, to distinguish the samples treated with ultrasound and microwave techniques from Jerez vinegar, the samples that showed the most significant impact from these treatments (US3 and MW2) were selected for sensory analysis. The sensory characteristics of fresh control, Jerez, US3 and MW2 samples, comprising appearance, aroma, flavor (sweetness, sourness, bitterness, pungency, and other specific descriptors), and overall impression, including a total of 38 attributes, are shown in Table 4.

Jerez vinegar is described by its amber tint with mahogany undertones, providing subtle notes of nuts, wood, and a clear acetic aroma. With a long duration of aging, it develops intricate hints of vanilla, nuts, and aged wood [53]. This study explores the sensory descriptors of accelerated aged Jerez samples, leading to the development of distinct lexicons [54]. Secondly, the impacts of ultrasound and microwave treatments have been investigated on the sensory characteristics of the fresh control sample. It was found that, in the appearance field, the darkest color, scoring 9.1 points, was observed in the traditionally aged Jerez sample. A dark color aligns with the color characteristics, indicating a high intensity of red absorbance measurement. The second highest color was observed for the US3 sample scoring 5.5 points and, as with the other analyses, ultrasound showed a stronger influence on the color of samples when compared with microwave. In the odor category of the US3 sample, overall intensity scored 2.3, vinegar ID scored 6.5, and winy character scored 2.4, results which were found to be closer to the Jerez sample than the others. Additionally, like the Jerez sample, raisin, alcohol/liquor, woody, spicy, clove, toasted, nuts, and leather lexicons were identified in the odor category of the US3 sample. Thanks to aging, many attributes exhibited a meaningful difference in the odor property of the samples that were traditionally aged and those which underwent accelerated aging. These differences, mainly with an increasing quantity from the fresh control, were observed in the attributes of raisin (0.6), liquor (0.1), woody (0.9), toasted (0.3), nuts (0.4), and leather (0.2). A change in the attributes of woody, toasted, and nut related to the aging of the samples taking place in wood casks [6]. Additionally, fruity notes were found as 3.1, 6.3, and 5.1 in the Jerez, US3 and MW2 samples, respectively. The vanilla attribute was found, with a score of 0.1 in odor for the MW2 sample, as with Jerez.

Following odor characterization, the flavor lexicons of Jerez showed close values to the US3 sample compared with MW2 sample. Overall flavor (6.6) and vinegar ID (6.57) in the US3 sample were perceived as the closest to those of the Jerez sample. Raisin, woody, spicy, and toasted attributes were identified with a low point of 0.1 in the US3 sample. Through the aging process, a decline in fruity taste (from 5.1 to 3.6), sourness (from 8.1 to 6.6), and pungent (from 4.1 to 2.5) profiles were determined between fresh control and US3 samples. The decrease in these attributes, particularly sourness and pungent profiles, may be attributed to change in acidic compounds, predominantly acetic acid substances [5,55]. The higher fruity profile in fresh vinegar samples is related to the presence of fresh fruit notes from grapes in the volatile compounds of ethyl esters and other acetates [56]. As can be seen in volatiles, a significant decrease in several ester components, namely, ethyl acetate, 2-methylbutyl acetate, and isobutyl acetate, affects the change in the concentration of fruity notes in the Jerez and US3 samples. An increase in the sweetness attribute was observed in all aged samples, with the highest value of 1.6 recorded in the US3 sample. The aftertaste attribute in the US3 sample was also found to be similar to that of the Jerez sample, with scores of 4.9 and 4.2, respectively.

As a conclusion, by evaluation of all of the attributes of the samples, the ultrasound-treated sample was found to be the closest one to the Jerez sample stem, based on the difference in volatile compounds. Although the treatment duration with wood chips was relatively short (only 10 h) compared with the traditional Jerez sample, which is aged for at least 6 months, the impact of the treatment was still noticeable in many sensory attributes. Based on the results, it can be stated that long-duration ultrasound treatment may significantly influence volatile components and sensory properties. An ultrasound effect on rice wine aging was also found, with a similar taste to the traditional system [57]. Although microwave treatment also influenced the sensory attributes of the sample, its impact was less pronounced than that of ultrasound. This may be explained by the shorter duration of the microwave treatment, which did not provide sufficient time to increase the collision rate or the diffusion rate of chemical substances [58]. The treatment duration was limited due to the rapid temperature rise in the liquid sample caused by the high power of the microwave. Therefore, in future studies, controlling the temperature may allow the application of different microwave power levels, which could lead to different outcomes. Thus, while the magnetic field is not widely recognized for accelerating the aging of fermented foods, ultrasound has shown promising progress in wine aging [46]. To the best of our knowledge, a sensory profile has been obtained via the comparison of a fresh control and Jerez under an accelerated aging process with ultrasound and microwave techniques, as have many new lexicons, particularly for odor and flavor profiles, which have in turn been analyzed and examined in this study.

### 3.5. PCA Analysis

**Figure 2:**
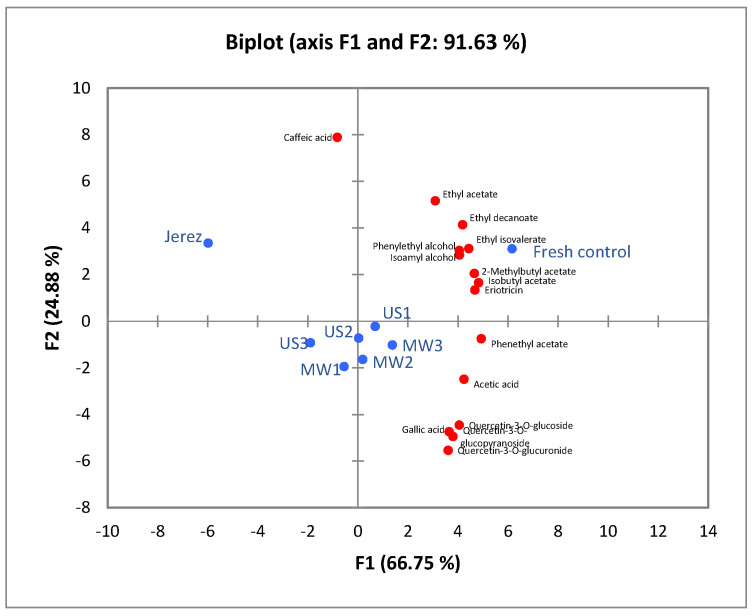
Principal component analysis (PCA) biplot (axes F1 and F2) showing the distribution of volatile and phenolic compounds and sample groups.

Principal component analysis (PCA) was applied to evaluate the relationships among the samples (fresh control, Jerez, US1, US2, US3 and MW1, MW2, MW3) based on their volatile and phenolic profiles. As shown in Figure 2, the first two principal components (PC1 and PC2) explained 91.63% of the total variance (66.75% and 24.88%, respectively), indicating a strong model representation. The eigenvalues and contribution values of the analyses are presented in Tables S2 and S3. The score plot revealed a clear separation between the fresh control and the treated samples, while US-treated and MW-treated samples were clustered closely, suggesting similar compositional characteristics. The fresh control samples were mainly associated with esters such as ethyl acetate, ethyl isovalerate, and 2-methylbutyl acetate, which contribute to fruity and floral aromas. In contrast, the treated samples (especially US3 and MW1–MW2) were more correlated with phenolic compounds, such as gallic acid and quercetin derivatives, indicating that processing affected the phenolic composition. As observed in Figure 2, all ultrasound-treated samples (US1–US3) are positioned closer to the Jerez sample compared with the microwave-treated ones. This clustering pattern suggests that ultrasound treatment caused changes in the volatile and phenolic composition that are more similar to those found in the traditionally aged Jerez sample. In contrast, the microwave-treated samples (MW1–MW3) remained closer to the central region of the plot, indicating a less pronounced alteration in their compositional profiles when compared with US treated samples. The Jerez sample appears distinctly separated, primarily influenced by higher levels of caffeic acid and phenylethyl alcohol. However, overall, the PCA results demonstrate that ultrasound and microwave treatments modified both volatile and phenolic profiles, leading to distinct clustering patterns when compared with the fresh control.

**Figure 3:**
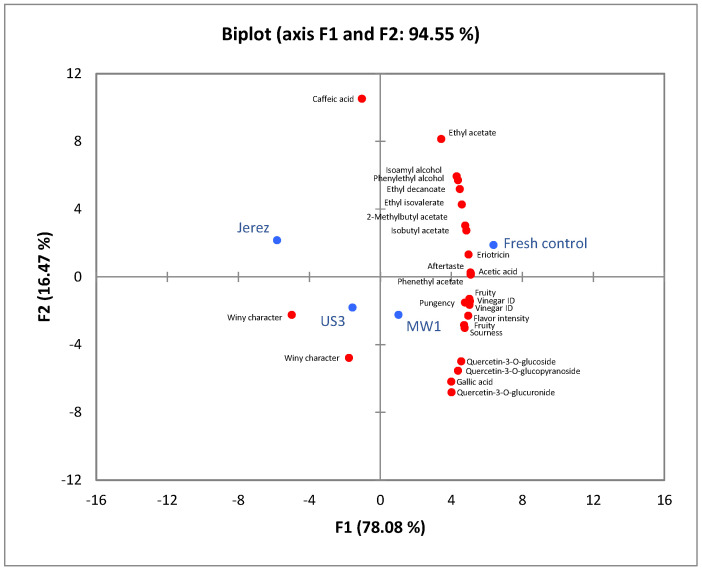
Principal component analysis (PCA) biplot (axes F1 and F2) showing the distribution of volatile, phenolic, sensory profiles and sample groups.

A second PCA was performed by integrating sensory attributes with volatile and phenolic compounds to explore the relationship between chemical composition and sensory perception as shown in Figure 3. In this analysis, the fresh control, Jerez, US3, and MW1 samples were included as representative groups used in the sensory evaluation to compare the influence of different treatments on both chemical and sensory characteristics. The first two principal components (PC1 and PC2) explained 94.55% of the total variance (78.08% and 16.47%, respectively), indicating a robust model. The eigenvalues and contribution values of the PCA are given in Tables S4 and S5. The score plot revealed distinct clustering among the samples. The fresh control was positioned on the positive side of PC1, closely associated with esters such as ethyl acetate, ethyl isovalerate, and isoamyl alcohol, as well as sensory descriptors like “fruity,” “flavor intensity,” and “vinegar identity,” reflecting its more pleasant and balanced aroma profile. In contrast, the ultrasound (US3) and microwave (MW1) samples were located closer to attributes such as “winy character,” “pungency,” and “aftertaste,” suggesting the development of more intense and less fresh sensory notes after processing. The Jerez sample appeared distinctly separated on the negative side of PC1, strongly correlated with caffeic acid and phenylethyl alcohol, which contribute to its mature and oxidized sensory character. As seen from the figure, the US3 sample is positioned closer to the Jerez sample, which was aged traditionally, suggesting that ultrasound treatment may induce compositional changes similar to those occurring during conventional aging. Consequently, the PCA indicates that ultrasound and microwave treatments modified both the chemical and sensory profiles, producing perceptible differences from the fresh control and highlighting the influence of processing on product quality. To summarize, these PCA results are consistent with the individual analyses of volatile and phenolic compounds, confirming that the observed clustering reflects the compositional differences identified in the chemical data.

## 4. Conclusions

The fresh control sample was subjected to the aging process with the accelerated non-thermal processing of the ultrasound and microwave techniques. After processing, to evaluate the consequences and impact of the alternative aging technologies on the samples, samples were subjected to thorough analysis encompassing physicochemical properties, chemical composition, and sensory attributes. To make an evaluation of the traditional methods, a Jerez sample, aged at least six months in oak barrels, was also analyzed and compared with the other samples. The ultrasound and microwave techniques were performed under three different parameters, as US1 (0.5 h), US2 (2 h) and US3 (10 h), and MW1 (640 W for 10 min), MW2 (640 W for 20 min), and MW3 (800 W for 10 min). Comparative analysis revealed a notable difference in phenolic, volatile compounds and sensory properties. For total phenolics, US1 treatment was found to be the closest one to the Jerez sample among the other ultrasound treatments, and also among all of the treatments, with a content of 3943 µg/kg. For microwave treatment, the 3988 µg/kg MW2 treatment was found to be the most effective among the other microwave treatments. A significant result was also obtained in total volatile compounds, with 623 mg/L obtained through US3 treatment and 716 mg/L with MW1 treatment. However, ultrasound still has the most impact on volatile components by showing the closest value to total phenolics in the Jerez sample, with 449 mg/L. The noteworthy differences stem from the volatile compounds, with primary components, namely esters (including ethyl acetate and 2-methylbutyl acetate), organic and carboxylic acids (i.e., acetic acid), and aldehydes (such as benzaldehyde), exhibiting a decrease in concentration over traditional and accelerated aging processes. Aldehydes and ketones also showed a declining trend by a short aging duration process.

The colorimetric properties of the samples were analyzed, though no significant changes were detected following ultrasound and microwave treatments. This may be due to the short aging duration, which did not allow sufficient interaction time between the wood chips and the sample. Among the treated samples, US3 was observed to be the closest to the Jerez sample, with yellow (69.7%), red (21.1%), and blue (9.2%) components. This similarity may be attributed to the limited effect of phenolic compounds during the short aging treatments. Furthermore, a comprehensive sensory evaluation has been conducted across all samples, revealing a substantial impact of accelerating aging on various sensory contributors. The samples most affected in terms of total volatile compounds—US3 and MW1—were selected for sensory analysis. Both exhibited differences from the fresh control sample, particularly in the intensity of woody, spicy, fruity, and nutty notes. Additionally, the US3 sample showed enhanced sensory characteristics with the presence of toast, liquor, and clove attributes. As a result, MW1 among the microwave treatments and US3 among the ultrasound treatments had the greatest influence on the accelerated aging of the samples. In particular, US3 was found to be the most effective treatment overall. This suggests that ultrasound may be more effective in this field due to the acoustic cavitation effect, which enhances chemical reactions and oxidation within just 10 h. In summary, this investigation demonstrated the significant impact of accelerated aging techniques on the fresh sample, resulting in a notable resemblance to the traditionally aged Jerez sample. The proposed methods could serve as a viable alternative to traditional aging and may also be applicable to other fermented and aged products.

## Figures and Tables

**Table 1:** Retention time, mass spectral characteristics, and concentration of phenolic compounds (µg kg^−1^) present in fresh control, *Jerez* and aged vinegars with US and MW techniques.

Chemical Family	Code	Compound	RT (min)	[M–H] (*m*/*z*)	MS/MS (*m*/*z*)	Fresh Control	Jerez	US1	US2	US3	MW1	MW2	MW3
Stilbenes	1	(E)-resveratrol	8.16	229	135.10/**107.10** ^†^/91.10	ND ^‡ d^	25.9 ± 0.4 ^a^	27.7 ± 0.3 ^a^	ND ^d^	21.4 ± 0.3 ^b^	15.3 ± 0.2 ^c^	ND ^d^	18.5 ± 0.6 ^b^
Flavones	2	Luteolin-7-*O*-glucoside	6.68	448.90	**287.10**/153.10/135.15	8.38 ± 0.4 ^b^	5.26 ± 0.3 ^c^	10.0 ± 0.2 ^a^	8.35 ± 0.3 ^b^	7.95 ± 0.3 ^b^	8.3 ± 0.2 ^b^	5.58 ± 0.1 ^c^	8.93 ± 0.3 ^b^
Flavanones	3	Eriotricin	5.95	595.20	**287.05**/150.09/135.05	65.8 ± 0.6 ^a^	ND ^c^	24.9 ± 0.2 ^b^	24.8 ± 0.2 ^b^	24.8 ± 0.3 ^b^	25.2 ± 0.4 ^b^	25.0 ± 0.3 ^b^	25.0 ± 0.2 ^b^
	4	Hesperidin	7.02	609.20	**301**/163.90/150.95	^¶^ tr ^b^	ND ^a^	ND ^a^	ND ^a^	ND ^a^	ND ^a^	ND ^a^	ND ^a^
Flavonols	5	Myricetin-3-*O*-glucoside	5.69	481.10	**319.10**/273.10/153	32.3 ± 0.3 ^a^	28.6 ± 0.2 ^a^	31.6 ± 0.2 ^a^	32.5 ± 0.5 ^a^	30.7 ± 0.3 ^a^	32.1 ± 0.4 ^a^	ND ^b^	32.7 ± 0.3 ^a^
	6	Quercetin-3-*O*-glucoside	5.95	463.25	**300.15**/271.15/255.20	232 ± 6.1 ^a^	60.2 ± 0.7 ^c^	234 ± 5.2 ^a^	210 ± 6.4 ^a^	175 ± 4.6 ^b^	224 ± 6.5 ^a^	217 ± 6.2 ^a^	230 ± 5.5 ^a^
	7	Quercetin-3-*O*-glucuronide	5.97	478.95	**303.05**/229/153/112.8	1806 ± 10 ^a^	1029 ± 7 ^c^	1768 ± 7 ^b^	1772 ± 9 ^b^	1738 ± 6 ^b^	1863 ± 15 ^a^	1852 ± 11 ^a^	1877 ± 15 ^a^
	8	Quercetin-3-*O*-galactoside	5.98	465	**303.10**/229.1/164/153	240 ± 2.1 ^a^	61.9 ± 0.8 ^c^	232 ± 1 ^b^	231 ± 3 ^b^	228 ± 2 ^b^	241 ± 2 ^a^	242 ± 2 ^a^	248 ± 3 ^a^
	9	Quercetin-3-*O*-rutinoside	6.20	608.90	301/**299.95**/270.95	54.5 ± 0.5 ^a^	ND ^b^	56.4 ± 0.4 ^a^	56.0 ± 0.5 ^a^	51.5 ± 0.3 ^b^	51.4 ± 0.4 ^b^	51.9 ± 0.3 ^b^	ND ^b^
	10	Quercetin-3-*O*-glucopyranoside	6.41	463.10	300.95/**300**/270.95	202 ± 4 ^a^	79.7 ± 0.7 ^d^	175 ± 1 ^b^	184 ± 2 ^b^	161 ± 2 ^c^	211 ± 4 ^a^	206 ± 2 ^a^	215 ± 3 ^a^
Phenolic acids and derivatives	11	Gallic acid	1.18	169.10	**124.95**/124.30/78.95	1191 ± 8 ^a^	642 ± 4 ^c^	1120 ± 7 ^b^	1180 ± 7 ^a^	1178 ± 7 ^a^	1133 ± 6 ^b^	1147 ± 6 ^b^	1109 ± 8 ^b^
	12	Chlorogenic acid	3.22	353.30	**191.05**/92.95/85.05	23.4 ± 0.3 ^b^	30.6 ± 0.3 ^a^	15.1 ± 0.2 ^c^	15.0 ± 0.2 ^c^	ND ^d^	14.8 ± 0.1 ^c^	15.2 ± 0.3 ^c^	15.0 ± 0.2 ^c^
	13	Caffeic acid	4.12	179.10	**135**/134/106.95	307 ± 7 ^a^	336 ± 8 ^a^	239 ± 2 ^b^	238 ± 2 ^b^	251 ± 7 ^b^	198 ± 2 ^d^	214 ± 3 ^d^	225 ± 2 ^c^
Anthocyanins	14	Peonidin-3,5-di-*O*-glucoside	4.89	625.15	462.95/**300.95**/285.95	ND ^b^	ND ^b^	ND ^b^	ND ^b^	ND ^b^	ND ^b^	11.9 ± 0.1 ^a^	ND ^b^
	15	Cyanidin-3-*O*-rutinoside	4.94	595.10	449/**287.10**	tr ^a^	ND ^a^	ND ^a^	ND ^a^	ND ^a^	ND ^a^	ND ^a^	ND ^a^
	16	Pelargonidin-3-*O*-glucoside	5.04	433	**271.10**/121.10	tr ^b^	15.3 ± 0.2 ^a^	ND ^b^	ND ^b^	tr ^b^	tr ^b^	tr ^b^	ND ^b^
	17	Peonidin-3-*O*-glucoside	5.14	463	**301.10**/286/201.05	ND ^a^	ND ^a^	ND ^a^	tr ^a^	ND ^a^	ND ^a^	ND ^a^	ND ^a^
	18	Malvidin-3-*O*-glucoside	5.16	493.10	**331.10**/315.10/287.10	tr ^a^	ND ^a^	ND ^a^	ND ^a^	ND ^a^	ND ^a^	ND ^a^	ND ^a^
	19	Malvidin-3-*O*-galactoside	5.21	493.20	**331.10**/315.10/287.10	tr ^a^	ND ^a^	ND ^a^	ND ^a^	ND ^a^	ND ^a^	ND ^a^	ND ^a^
		Total of polyphenolic compounds				4230 ^a^	2515 ^d^	3943 ^c^	3951 ^c^	3967 ^c^	4016 ^b^	3988 ^c^	4034 ^b^

^†^ The *m*/*z* values of the dominant ions are highlighted using bold font. ^¶^ tr: trace. ^‡^ ND: not detected. Mean values obtained from the duplicate analysis of all vinegar samples. Values followed by the same letter, within the same row, were not significantly different (*p* > 0.05), according to Tukey’s least significant difference test.

**Table 2:** Volatile components identified in fresh control, *Jerez* and aged vinegars with US and MW techniques (mg L^−1^).

Chemical Family	Compound	Code	RT (min) ^‡^	RI ^†^	Fresh Control	Jerez	US1	US2	US3	MW1	MW2	MW3
Esters	Methyl acetate	V1	2.80	857	3.76 ± 0.2 ^a^	1.36 ± 0.2 ^d^	2.51 ± 0.2 ^c^	2.51 ± 0.05 ^c^	2.30 ± 0.2 ^c^	2.37 ± 0.2 ^c^	2.54 ± 0.3 ^c^	3.24 ± 0.4 ^b^
	Ethyl acetate	V2	3.17	888	43.9 ± 2.1 ^a^	32.5 ± 1.5 ^c^	38.6 ± 1.7 ^b^	32.4 ± 0.9 ^c^	25.9 ± 1.8 ^d^	29.5 ± 1.2 ^c^	29.8 ± 1.1 ^c^	30.2 ± 1.5 ^c^
	Ethyl propanoate	V5	3.89	947	0.08 ± 0.01 ^b^	0.17 ± 0.02 ^a^	0.06 ± 0.02 ^b^	0.07 ± 0.01 ^b^	0.06 ± 0.01 ^b^	0.05 ± 0.01 ^b^	0.05 ± 0.01 ^b^	0.06 ± 0.01 ^b^
	Ethyl isobutyrate	V6	4.01	957	0.33 ± 0.03 ^a^	0.15 ± 0.01 ^b^	0.20 ± 0.02 ^b^	0.19 ± 0.01 ^b^	0.15 ± 0.02 ^b^	0.16 ± 0.01 ^b^	0.17 ± 0.02 ^b^	0.19 ± 0.01 ^b^
	Isobutyl acetate	V7	4.71	1007	12.3 ± 0.2 ^a^	3.99 ± 0.4 ^c^	8.01 ± 0.7 ^b^	7.60 ± 0.2 ^b^	6.36 ± 0.4 ^b^	6.32 ± 0.5 ^b^	6.82 ± 0.8 ^b^	7.98 ± 0.2 ^b^
	Methyl isovalerate	V8	4.82	1012	0.24 ± 0.06 ^a^	0.09 ± 0.01 ^c^	0.14 ± 0.01 ^b^	0.14 ± 0.04 ^b^	0.14 ± 0.04 ^b^	0.11 ± 0.02 ^b^	0.12 ± 0.04 ^b^	0.13 ± 0.01 ^b^
	Ethyl 2-methylbutanoate	V9	5.46	1041	0.19 ± 0.01 ^a^	0.08 ± 0.01 ^c^	0.14 ± 0.01 ^b^	0.11 ± 0.01 ^b^	0.09 ± 0.02 ^c^	0.09 ± 0.01 ^c^	0.09 ± 0.01 ^c^	0.12 ± 0.02 ^b^
	Ethyl isovalerate	V10	5.78	1056	5.33 ± 0.7 ^a^	1.78 ± 0.1 ^b^	3.59 ± 0.4 ^b^	2.85 ± 0.1 ^b^	2.46 ± 0.4 ^b^	2.28 ± 0.05 ^b^	2.42 ± 0.4 ^b^	2.89 ± 0.3 ^b^
	2-Methylbutyl acetate	V13	7.22	1113	125 ± 1 ^a^	36.6 ± 3.9 ^d^	85.7 ± 6.1 ^b^	74.7 ± 2.4 ^b^	61.6 ± 6.4 ^c^	58.2 ± 4.6 ^c^	62.2 ± 6.1 ^c^	71.5 ± 4.3 ^b^
	Ethyl hexanoate	V15	11.45	1225	1.84 ± 0.1 ^a^	0.32 ± 0.02 ^e^	1.06 ± 0.2 ^b^	0.95 ± 0.03 ^b^	0.79 ± 0.1 ^d^	0.71 ± 0.06 ^d^	0.76 ± 0.1 ^d^	0.86 ± 0.08 ^c^
	Hexyl acetate	V16	13.22	1262	0.81 ± 0.1 ^c^	0.94 ± 0.1 ^c^	0.46 ± 0.2 ^d^	0.92 ± 0.2 ^c^	1.48 ± 0.2 ^b^	1.65 ± 0.1 ^b^	2.31 ± 0.1 ^a^	2.61 ± 0.3 ^a^
	2-Methylbutyl isovalerate	V18	14.42	1288	1.34 ± 0.1 ^a^	0.19 ± 0.06 ^d^	0.57 ± 0.1 ^b^	0.50 ± 0.06 ^b^	0.37 ± 0.1 ^c^	0.35 ± 0.04 ^c^	0.25 ± 0.04 ^d^	0.42 ± 0.02 ^c^
	Acetoin acetate	V19	18.79	1370	0.52 ± 0.06 ^a^	0.28 ± 0.06 ^d^	0.32 ± 0.1 ^c^	0.38 ± 0.04 ^c^	0.33 ± 0.02 ^c^	0.38 ± 0.08 ^c^	0.44 ± 0.05 ^b^	0.48 ± 0.08 ^a^
	Ethyl decanoate	V25	32.78	1630	5.08 ± 0.5 ^a^	0.29 ± 0.1 ^d^	1.22 ± 0.07 ^b^	1.00 ± 0.1 ^b^	0.53 ± 0.08 ^c^	0.78 ± 0.1 ^c^	0.78 ± 0.05 ^c^	1.14 ± 0.09 ^b^
	Diethyl succinate	V27	34.31	1664	0.40 ± 0.09 ^b^	0.79 ± 0.03 ^a^	0.24 ± 0.08 ^d^	0.23 ± 0.04 ^d^	0.24 ± 0.03 ^d^	0.20 ± 0.03 ^d^	0.22 ± 0.05 ^d^	0.31 ± 0.08 ^c^
	Benzyl acetate	V28	36.21	1706	0.67 ± 0.1 ^a^	0.36 ± 0.01 ^d^	0.34 ± 0.08 ^d^	0.42 ± 0.08 ^c^	0.30 ± 0.04 ^d^	0.41 ± 0.02 ^c^	0.32 ± 0.02 ^d^	0.51 ± 0.08 ^b^
	Methyl salicylate	V29	37.76	1745	0.20 ± 0.01 ^a^	0.25 ± 0.04 ^a^	0.15 ± 0.05 ^b^	0.14 ± 0.02 ^b^	0.12 ± 0.01 ^b^	0.17 ± 0.01 ^b^	0.17 ± 0.04 ^b^	0.20 ± 0.03 ^a^
	Ethyl benzeneacetate	V30	38.60	1765	0.27 ± 0.05 ^a^	0.13 ± 0.02 ^c^	0.13 ± 0.02 ^c^	0.13 ± 0.01 ^c^	0.13 ± 0.01 ^c^	0.12 ± 0.02 ^c^	0.15 ± 0.01 ^c^	0.16 ± 0.03 ^b^
	Phenethyl acetate	V31	39.70	1792	27.5 ± 0.8 ^a^	7.87 ± 0.4 ^d^	19.3 ± 0.4 ^b^	18.1 ± 0.3 ^b^	15.3 ± 0.8 ^c^	18.1 ± 0.7 ^b^	18.8 ± 0.9 ^b^	21.5 ± 1.1 ^b^
	Isopropyl myristate	V36	48.24	2010	0.41 ± 0.07 ^c^	0.22 ± 0.03 ^d^	0.66 ± 0.06 ^b^	0.97 ± 0.1 ^a^	0.30 ± 0.1 ^d^	0.23 ± 0.09 ^d^	0.28 ± 0.07 ^d^	0.12 ± 0.03 ^d^
Acids	Acetic acid	V21	21.9	1427	716 ± 20 ^a^	314 ± 6.6 ^d^	659 ± 15 ^a^	597 ± 18 ^b^	451 ± 16 ^c^	533 ± 1.8 ^b^	715 ± 25 ^a^	716 ± 28 ^a^
	Isobutyric acid	V24	29.0	1554	0.95 ± 0.1 ^b^	0.92 ± 0.03 ^b^	1.18 ± 0.6 ^b^	1.03 ± 0.04 ^b^	1.06 ± 0.1 ^b^	1.13 ± 0.1 ^b^	1.23 ± 0.08 ^a^	1.32 ± 0.2 ^a^
	Isovaleric acid	V26	33.87	1654	26.8 ± 1.2 ^a^	18.2 ± 1.1 ^b^	21.6 ± 1.5 ^b^	25.4 ± 1.8 ^a^	23.1 ± 1.5 ^b^	28.0 ± 2.0 ^a^	30.5 ± 3.1 ^a^	32.8 ± 2.5 ^a^
	Hexanoic acid	V32	41.07	1828	0.72 ± 0.02 ^a^	0.33 ± 0.03 ^d^	0.34 ± 0.06 ^d^	0.46 ± 0.1 ^c^	0.50 ± 0.09 ^c^	0.55 ± 0.08 ^c^	0.68 ± 0.09 ^b^	0.75 ± 0.01 ^a^
	Octanoic acid	V37	48.64	2013	1.10 ± 0.1 ^a^	0.41 ± 0.01 ^d^	0.62 ± 0.1 ^c^	0.80 ± 0.1 ^b^	0.85 ± 0.1 ^b^	1.12 ± 0.1 ^a^	1.12 ± 0.2 ^a^	1.21 ± 0.2 ^a^
	Decanoic acid	V39	54.35	1386	0.28 ± 0.06 ^a^	0.07 ± 0.01 ^c^	0.17 ± 0.01 ^b^	0.20 ± 0.01 ^b^	0.19 ± 0.02 ^b^	0.26 ± 0.02 ^a^	0.25 ± 0.01 ^a^	0.28 ± 0.02 ^a^
Alcohols	Ethanol	V4	3.58	922	4.17 ± 0.5 ^a^	2.91 ± 0.1 ^b^	3.04 ± 0.03 ^b^	2.87 ± 0.1 ^c^	2.53 ± 0.3 ^c^	2.91 ± 0.05 ^b^	3.19 ± 0.4 ^b^	4.32 ± 0.7 ^a^
	Isobutyl alcohol	V11	6.44	1085	1.49 ± 0.1 ^a^	0.71 ± 0.04 ^d^	0.88 ± 0.1 ^c^	0.91 ± 0.03 ^c^	0.81 ± 0.07 ^c^	0.93 ± 0.1 ^b^	0.98 ± 0.08 ^b^	1.13 ± 0.02 ^b^
	Isoamyl alcohol	V14	10.26	1200	15.5 ± 1.3 ^a^	10.2 ± 1.2 ^b^	10.4 ± 1.3 ^b^	11.2 ± 1.3 ^b^	9.36 ± 0.4 ^c^	10.7 ± 1.0 ^b^	11.4 ± 1.0 ^b^	13.5 ± 0.8 ^a^
	Benzyl alcohol	V33	41.97	1852	0.42 ± 0.03 ^a^	0.38 ± 0.05 ^b^	0.26 ± 0.03 ^c^	0.21 ± 0.05 ^c^	0.20 ± 0.01 ^c^	0.26 ± 0.03 ^c^	0.24 ± 0.07 ^c^	0.30 ± 0.04 ^b^
	Phenylethyl alcohol	V34	43.22	1885	12.7 ± 0.8 ^a^	7.96 ± 0.1 ^c^	7.93 ± 0.5 ^c^	8.32 ± 0.6 ^c^	7.67 ± 0.6 ^c^	8.36 ± 0.6 ^c^	9.32 ± 0.7 ^b^	10.4 ± 0.8 ^b^
	Dodecyl alcohol	V35	45.74	1957	0.17 ± 0.02 ^a^	0.19 ± 0.03 ^a^	0.12 ± 0.03 ^b^	0.15 ± 0.05 ^b^	0.14 ± 0.03 ^b^	0.23 ± 0.06 ^a^	0.23 ± 0.05 ^a^	0.22 ± 0.03 ^a^
Aldehydes	Isovaleraldehyde	V3	3.45	911	0.87 ± 0.02 ^a^	0.04 ± 0.01 ^d^	0.25 ± 0.07 ^c^	0.18 ± 0.01 ^c^	0.19 ± 0.02 ^c^	0.19 ± 0.03 ^c^	0.20 ± 0.01 ^c^	0.42 ± 0.04 ^b^
	Furfural	V22	22.97	1445	0.56 ± 0.07 ^a^	0.36 ± 0.01 ^b^	0.83 ± 0.03 ^c^	0.89 ± 0.08 ^c^	0.85 ± 0.03 ^c^	0.78 ± 0.08 ^c^	0.90 ± 0.08 ^c^	1.00 ± 0.07 ^c^
	Benzaldehyde	V23	25.95	1496	2.52 ± 0.3 ^a^	0.23 ± 0.06 ^d^	1.48 ± 0.2 ^c^	1.46 ± 0.2 ^c^	1.18 ± 0.04 ^c^	1.48 ± 0.2 ^c^	1.41 ± 0.2 ^c^	1.70 ± 0.06 ^b^
Ketones	Acetoin	V17	13.46	1268	3.54 ± 0.5 ^a^	2.56 ± 0.1 ^c^	2.19 ± 0.4 ^c^	2.31 ± 0.07 ^c^	2.01 ± 0.1 ^c^	2.37 ± 0.07 ^c^	2.52 ± 0.5 ^c^	3.01 ± 0.3 ^b^
	2-Nonanone	V20	19.08	1375	0.47 ± 0.03 ^a^	0.11 ± 0.01 ^c^	0.19 ± 0.03 ^b^	0.19 ± 0.05 ^b^	0.16 ± 0.05 ^b^	0.14 ± 0.03 ^c^	0.15 ± 0.02 ^b^	0.16 ± 0.02 ^b^
Phenolic compounds	2-Ethylphenol	V38	52.13	2047	0.37 ± 0.01 ^a^	0.27 ± 0.01 ^b^	0.16 ± 0.01 ^c^	0.16 ± 0.01 ^c^	0.13 ± 0.01 ^d^	0.16 ± 0.01 ^c^	0.19 ± 0.01 ^c^	0.25 ± 0.01 ^b^
Others	Linalool 3,7-oxide	V12	6.83	1102	0.90 ± 0.01 ^a^	0.17 ± 0.01 ^d^	0.56 ± 0.01 ^b^	0.44 ± 0.01 ^b^	0.37 ± 0.01 ^c^	0.31 ± 0.01 ^c^	0.32 ± 0.01 ^c^	0.47 ± 0.01 ^b^
Total		*N = 39*			1019 ^a^	449 ^e^	876 ^c^	799 ^c^	623 ^d^	716 ^d^	910 ^b^	950 ^a^

^‡^ RT: Retention times on DB-Wax column. ^†^ RI: Retention index on DB-Wax column. Mean values obtained from the triplicate analysis of all vinegar samples. Values followed by the same letter, within the same row, were significantly different (*p* < 0.05), according to Tukey’s least significant difference test.

**Table 3:** The chromatic characteristics of fresh control, *Jerez* and aged vinegars with US and MW techniques.

Vinegar Type	A_420_^¶^	A_520_^¶^	A_620_^¶^	Color Intensity	Tonality	Color Density	Y ^¶^ (%)	R ^¶^ (%)	B ^¶^ (%)
Fresh control	1.031	0.305	0.091	1.43 ± 0.05 ^c^	3.39 ± 0.02 ^a^	1.34 ± 0.02 ^c^	72.3 ± 0.1 ^a^	21.4 ± 0.1 ^b^	6.3 ± 0.1 ^c^
Jerez	2.049	0.784	0.294	3.13 ± 0.07 ^a^	2.62 ± 0.07 ^b^	2.83 ± 0.05 ^a^	65.5 ± 0.1 ^b^	25.1 ± 0.1 ^a^	9.4 ± 0.1 ^a^
US1	1.085	0.334	0.108	1.53 ± 0.02 ^c^	3.25 ± 0.08 ^a^	1.42 ± 0.03 ^c^	71.1 ± 0.1 ^a^	21.9 ± 0.1 ^b^	7.0 ± 0.1 ^b^
US2	1.124	0.346	0.124	1.58 ± 0.03 ^c^	3.22 ± 0.08 ^a^	1.46 ± 0.08 ^b^	70.3 ± 0.1 ^a^	21.9 ± 0.1 ^b^	7.8 ± 0.1 ^b^
US3	1.253	0.382	0.160	1.82 ± 0.05 ^b^	3.31 ± 0.09 ^a^	1.65 ± 0.10 ^b^	69.7 ± 0.1 ^a^	21.1 ± 0.1 ^b^	9.2 ± 0.1 ^a^
MW1	1.047	0.313	0.092	1.45 ± 0.02 ^c^	3.35 ± 0.11 ^a^	1.36 ± 0.07 ^c^	72.2 ± 0.1 ^a^	21.5 ± 0.1 ^b^	6.3 ± 0.1 ^c^
MW2	1.078	0.319	0.098	1.50 ± 0.03 ^c^	3.38 ± 0.10 ^a^	1.40 ± 0.06 ^c^	72.1 ± 0.1 ^a^	21.3 ± 0.1 ^b^	6.6 ± 0.1 ^c^
MW3	1.055	0.314	0.094	1.46 ± 0.02 ^c^	3.37 ± 0.15 ^a^	1.37 ± 0.05 ^c^	72.2 ± 0.1 ^a^	21.4 ± 0.1 ^b^	6.4 ± 0.1 ^c^

^¶^ Y: Yellow color. R: Red color. and B: Blue color. A_420_: Absorbance at 420 nm. A_520_: Absorbance at 520 nm. A_620_: Absorbance at 620 nm. Values followed by the same letter, within the same column, were significantly different (*p* < 0.05), according to Tukey’s least significant difference test.

**Table 4:** Descriptive sensory analysis of fresh control, Jerez and aged vinegars with US and MW techniques.

Attribute	ANOVA	Sample			
		Fresh Control	Jerez	US3	MW1
Appearance					
Color	***	4.4 ^c^	9.1 ^a^	5.5 ^b^	4.1 ^c^
*Untuoso* (texture)	^ǂ^ NS	4.4 ^a^	4.9 ^a^	4.4 ^a^	4.4 ^a^
Odor					
Odor intensity	***	2.9 ^a^	1.8 ^bc^	2.3 ^ab^	2.6 ^b^
Vinegar ID	***	8.0 ^a^	5.1 ^d^	6.5 ^c^	6.9 ^b^
Winy character	***	1.4 ^b^	2.7 ^a^	2.4 ^a^	2.2 ^a^
Raisin	***	0.0 ^c^	0.6 ^a^	0.6 ^a^	0.3 ^b^
Ethyl acetate	***	0.0 ^a^	0.1 ^a^	0.2 ^a^	0.1 ^a^
Alcohol/liquor	***	0.0 ^b^	0.3 ^a^	0.1 ^b^	0.0 ^b^
Woody	***	0.0 ^c^	0.9 ^a^	0.9 ^a^	0.4 ^b^
Fruity	***	6.3 ^a^	3.1 ^c^	5.3 ^b^	5.1 ^b^
Spicy	**	0.0 ^b^	0.2 ^b^	0.7 ^a^	0.1 ^b^
Vanilla	NS	0.0 ^a^	0.1 ^a^	0.0 ^a^	0.1 ^a^
Clove	**	0.0 ^b^	0.1 ^b^	0.4 ^a^	0.0 ^b^
Toasted	***	0.0 ^b^	0.4 ^a^	0.3 ^a^	0.1 ^b^
Nuts	**	0.0 ^b^	0.1 ^b^	0.4 ^a^	0.0 ^b^
Leather/old	**	0.0 ^b^	0.4 ^a^	0.2 ^a^	0.1 ^b^
Defects	NS	0.0 ^b^	0.3 ^a^	0.0 ^b^	0.0 ^b^
Flavor					
Flavor intensity	***	8.0 ^a^	5.6 ^c^	6.6 ^b^	7.5 ^ab^
Vinegar ID	***	8.1 ^a^	5.6 ^c^	6.6 ^b^	7.4 ^a^
Winy character	***	2.7 ^b^	2.9 ^b^	3.9 ^a^	2.7 ^b^
Raisin	NS	0.0 ^a^	0.2 ^a^	0.1 ^a^	0.0 ^a^
Ethyl acetate	NS	0.0 ^a^	0.0 ^a^	0.0 ^a^	0.0 ^a^
Alcohol/liquor	NS	0.0 ^a^	0.0 ^a^	0.0 ^a^	0.0 ^a^
Woody	**	0.0 ^b^	0.4 ^a^	0.1 ^b^	0.0 ^b^
Fruity	***	5.1 ^a^	2.8 ^c^	3.6 ^bc^	4.46 ^b^
Spicy	NS	0.0 ^a^	0.1 ^a^	0.1 ^a^	0.0 ^a^
Vanilla	NS	0.0 ^a^	0.0 ^a^	0.0 ^a^	0.1 ^a^
Clove	NS	0.0 ^a^	0.0 ^a^	0.0 ^a^	0.0 ^a^
Toasted	NS	0.0 ^a^	0.1 ^a^	0.1 ^a^	0.0 ^a^
Nuts	**	0.5 ^a^	0.3 ^b^	0.3 ^b^	0.4 ^a^
Leather/old	NS	0.0 ^a^	0.0 ^a^	0.0 ^a^	0.0 ^a^
Sweetness	***	1.1 ^b^	1.6 ^a^	1.6 ^a^	1.1 ^b^
Sourness	***	8.1 ^a^	5.6 ^c^	6.6 ^b^	7.9 ^a^
Bitterness	NS	0.4 ^a^	0.4 ^a^	0.4 ^a^	0.4 ^a^
Astringency	NS	0.0 ^a^	0.0 ^a^	0.0 ^a^	0.0 ^a^
Pungency	***	4.1 ^a^	2.0 ^b^	2.5 ^b^	3.8 ^a^
Defects	NS	0.0 ^a^	0.0 ^a^	0.0 ^a^	0.0 ^a^
Global					
Aftertaste	***	6.5 ^a^	4.3 ^c^	4.9 ^c^	5.6 ^ab^

Values (mean of 7 trained panelists) followed by the same letter. ^ǂ^ NS: Not significant at *p* < 0.05; **, ***, significant at *p* < 0.01, and *p* < 0.001, respectively.

## Data Availability

The original contributions presented in this study are included in the article/Supplementary Materials. Further inquiries can be directed to the corresponding author.

## Supplementary Materials

The following supporting information can be downloaded at https://www.mdpi.com/article/10.3390/foods14213665/s1, Figure S1: The preconditioning setup of oak chips before treatment; Figure S2: The ice machine (a), ultrasonic bath, and system setup (b); Figure S3: The infrared image of the sample bottle during MW1 treatment after 10 min; Figure S4: The infrared image of the sample bottle during MW2 treatment after 20 min (B); Figure S5: The infrared image of the sample bottle during MW3 treatment after 10 min; Figure S6: Set-up of samples for sensory analysis. Table S1: Statistical parameters for the quantitative determination of phenolic compounds, including calibration model equations, coefficients of determination (R^2^), relative standard deviations (RSD), and limits of detection (LOD) title; Table S2: Eigenvalues of Principal Component Analysis (PCA) for volatile and phenolic components; Table S3: Percentage contribution of volatile and phenolic variables in PCA; Table S4: Eigenvalues of Principal Component Analysis (PCA) for volatile, phenolic, and sensory componentstitle; Table S5: Percentage contribution of volatile, phenolic, and sensory variables in PCA.
