# Supplementary material for: Exploring Ultrasound and Microwave-Assisted Accelerated Aging of Jerez Vinegar: Impacts on Phenolic, Volatile, Colorimetric, and Sensory Properties
Source: Foods. 2025 Oct 27;14(21):3665. doi: 10.3390/foods14213665 (PMC12610971; doi:10.3390/foods14213665)
Supplement: Supplementary file 1 [file foods-14-03665-s001.zip › foods-3930455-supplementary.pdf]

SUPPLEMANTARY FILE

Exploring Ultrasound and Microwave-Assisted Accelerated Aging of Jerez Vinegar: Impacts on Phenolic, Volatile, Colorimetric, and Sensory Properties

Reyhan Selin Uysal [1,*], Hanán Issa-Issa [2], Ángel A. Carbonell-Barrachina [2] and Esther Sendra [2]

[1] Department of Genetics and Bioengineering, Istanbul Bilgi University, Istanbul 34060, Turkey; selin.uysal@bilgi.edu.tr

[2] Grupo de investigación "Calidad y Seguridad Alimentaria", Centro de Investigación e Innovación Agroalimentaria y Agroambiental (CIAGRO-UMH), Universidad Miguel Hernández de Elche, Carretera de Beniel, km 3.2, 03312-Orihuela, Alicante, Spain; hissa@umh.es ; angel.carbonell@umh.es; esther.sendra@umh.es

* Correspondence: selin.uysal@bilgi.edu.tr ; Tel.: (+902123116541)

Academic Editor: Firstname Lastname

Received: date
Revised: date
Accepted: date
Published: date

Citation: To be added by editorial staff during production.

Copyright: © 2025 by the authors. Submitted for possible open access publication under the terms and conditions of the Creative Commons Attribution (CC BY) license (https://creativecommons.org/licenses/by/4.0/).

Figure S1. The preconditioning setup of oak chips before treatment.

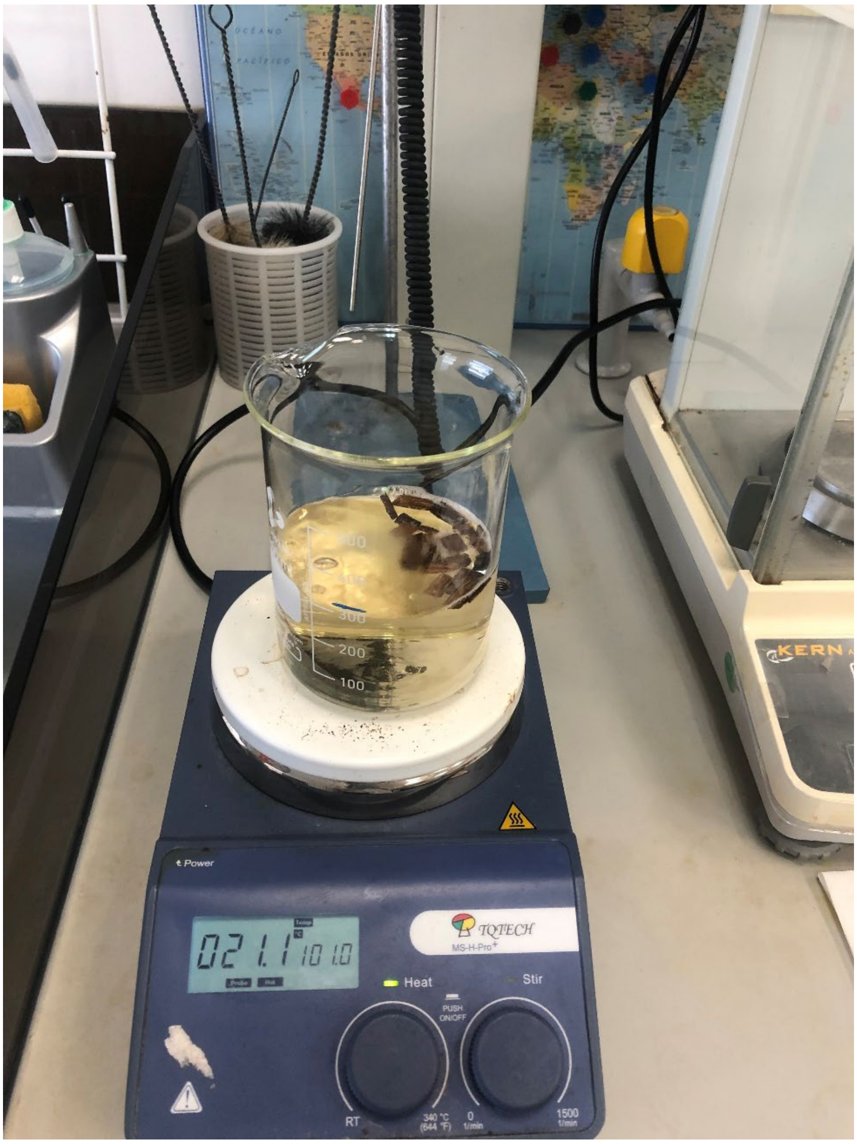

Figure S2. The ice machine (a), ultrasonic bath, and system setup (b).

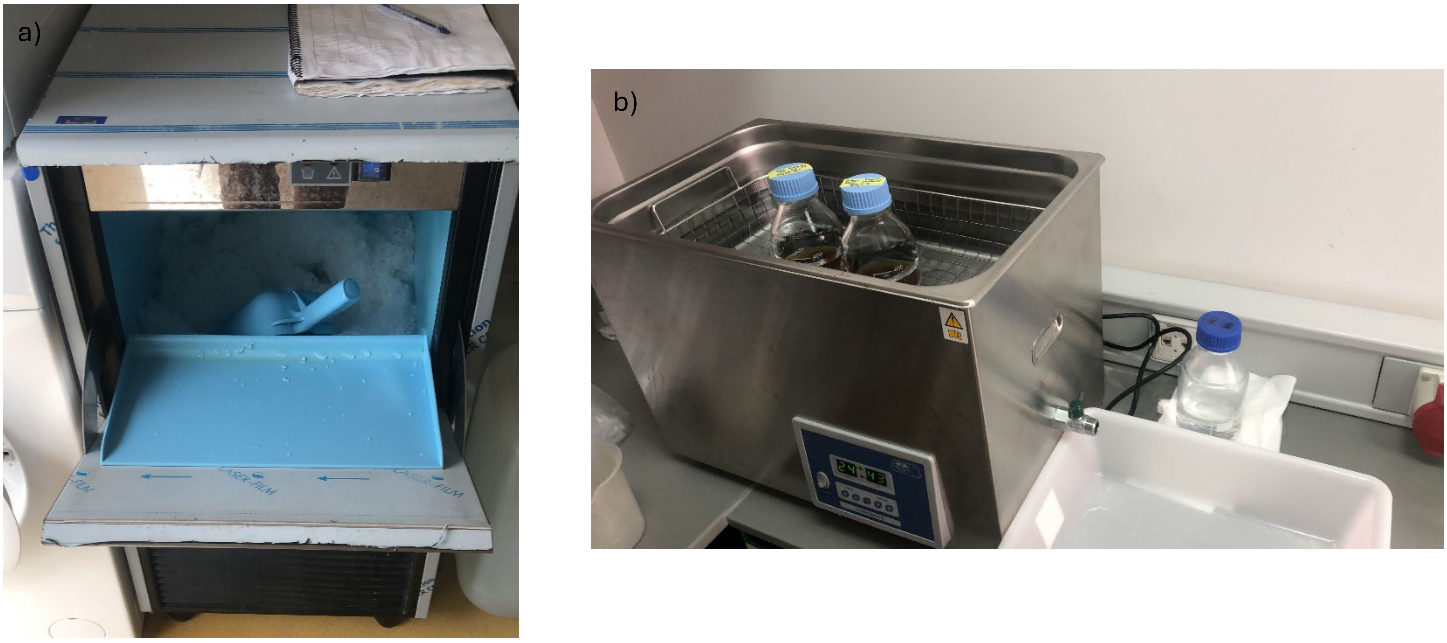

Figure S3. The infrared image of the sample bottle during M1 treatment after 10 min.

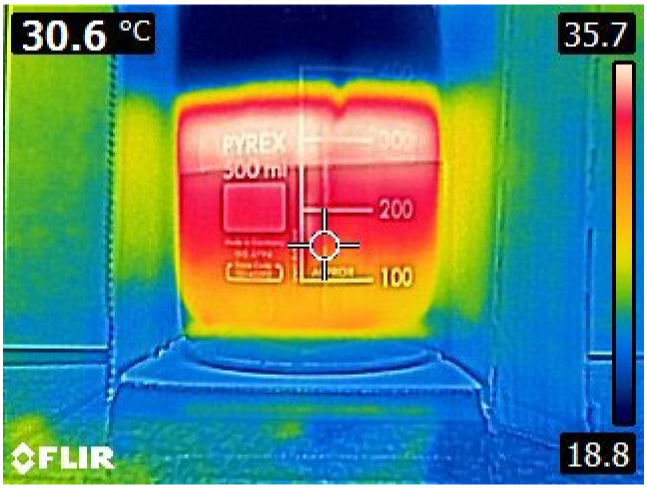

Figure S4. The infrared image of the sample bottle during M2 treatment after 20 min.

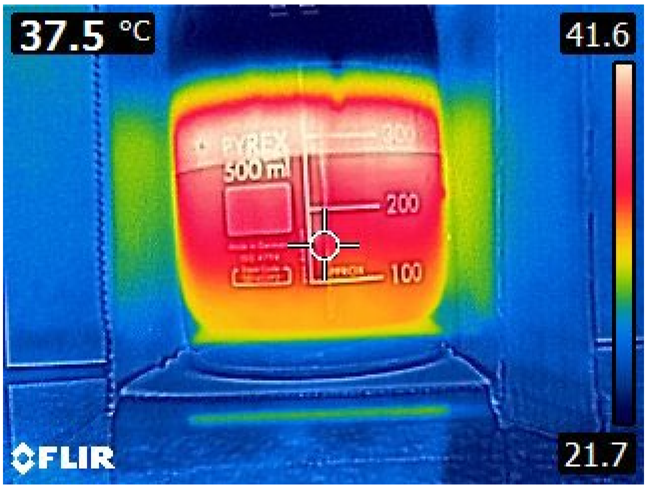

Figure S5. The infrared image of the sample bottle during M3 treatment after 10 min.

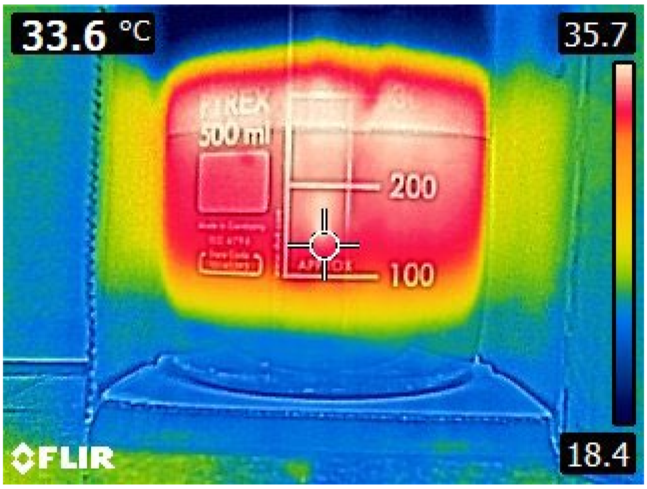

Figure S6. Set-up of samples for sensory analysis.

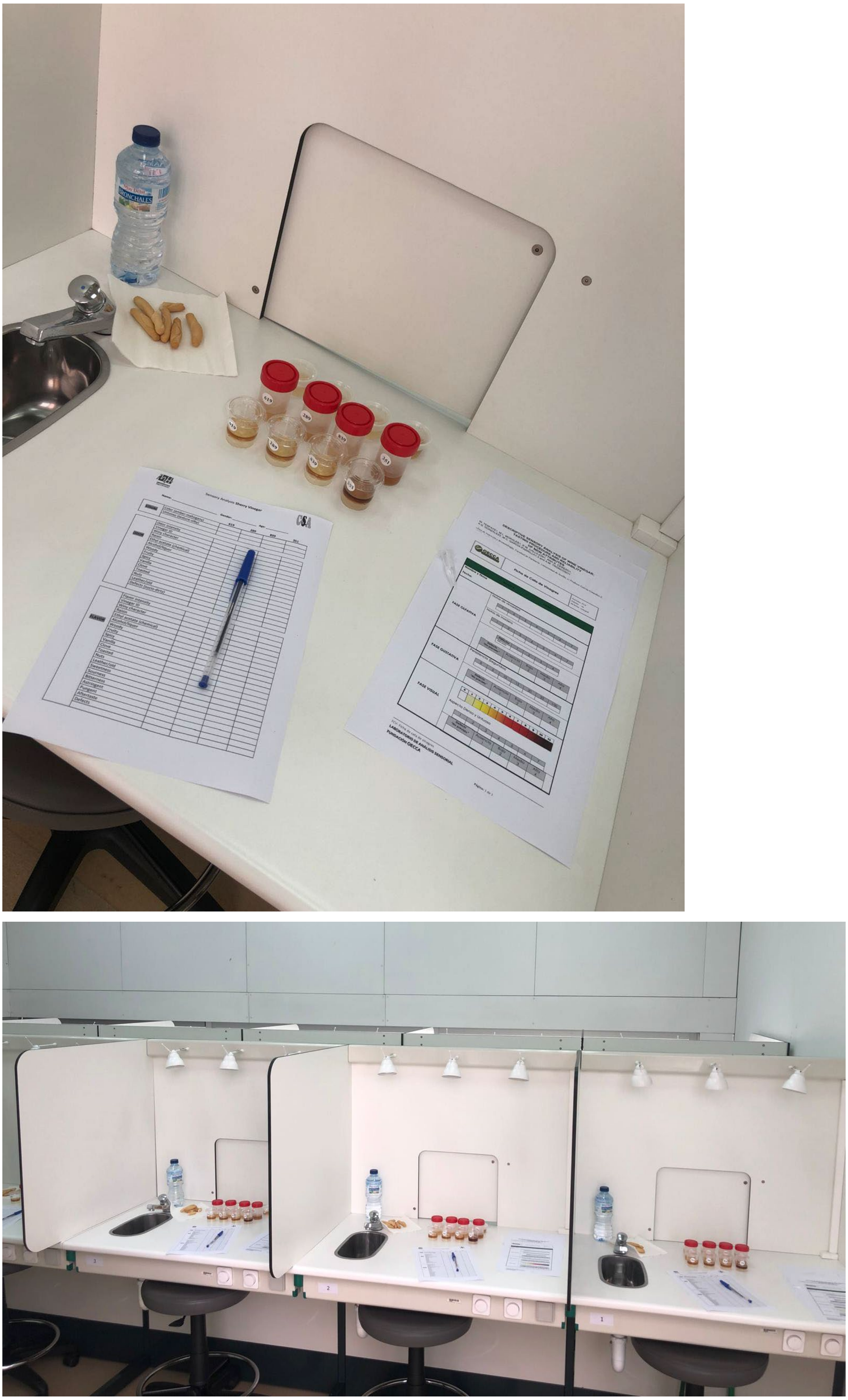

Table S1. Statistical parameters for the quantitative determination of phenolic compounds, including calibration model equations, coefficients of determination (R^2), relative standard deviations (RSD), and limits of detection (LOD)

Quercetin-3-O-Galactoside<br>Y=(8.59733e+006)X + (-679903)<br>r^2=0.9979<br>%RSD=8.4641<br>Detection Limit:0.001	Myricetin-3-O-Glucoside<br>Y=(2.90682e+006)X + (-368897)<br>r^2=0.9954<br>%RSD=28.8034<br>Detection Limit:0.01
Quercetin-3-O-Glucuronide<br>Y= (3.76908e+006)X + (-1.03231e+006<br>r^2=0.9955<br>%RSD=37.8961	Quercetin-3-O-Glucoside<br>Y=(726387)X + (-24920.4)<br>r^2=0.9974<br>%RSD=12.1271<br>Detection Limit:0.01
Quercetin-3-O-Rutinoside<br>Y=(203276)X+(-49882.9)<br>r^2=0.9978<br>%RSD=13.1895<br>Detection Limit:0.001	Quercetin-3-O-Glucopyranoside<br>Y=(2.27124e+006)X + (57945.6)<br>r^2=0.9999<br>%RSD=4.3268<br>Detection Limit:0.001
Peonidin-3,5-Di-O-Glucoside<br>Y=(8.29372e+006)X+ (-476886)<br>r^2=0.9978<br>%RSD=13.1176<br>Detection Limit: 0.001	Cyanidin-3-O-Rutinoside<br>Y=(2.53966e+007)X+ (1.13196e+006)<br>r^2=0.9944<br>%RSD=11.4749<br>Detection Limit: 0.01
Pelargonidin-3-O-Glucoside<br>Y=(2.00032e+007)X + (989672)<br>r^2=0.9964<br>%RSD=10.4504<br>Detection Limit:0.001	Peonidin-3-O-Glucoside<br>Y=(1.36252e+007)X+(413277)<br>r^2=0.9946<br>%RSD=8.5190<br>Detection Limit:0.02

Malvidin-3-O-Glucoside<br>Y=(3.64401e+007)X+(897291)<br>r^2=0.9992<br>%RSD=5.3522<br>Deteccion Limit: 0.001	Malvidin-3-O-Galactoside<br>Y=(2.55423e+007)X+(624029)<br>r^2=0.9993<br>%RSD=5.7028<br>Detection Limit:0.001
Eriotricin<br>Y=(2.63552e+006)X+(-314784)<br>r^2=0.9992<br>%RSD=7.0886<br>Detection Limit: 0.001	Hesperidin<br>Y=(766635)X + (57613.1)<br>r^2=0.96822<br>%RSD=45.1163<br>Detection Limit:0.001
Gallic acid<br>Y=(1.13997e+006)X+(67192.3)<br>r^2=0.9989<br>%RSD=6.3446<br>Detection Limit:0.01	Caffeic Acid<br>Y=(3.76791e+006)X+ (-134583)<br>r^2=0.9997<br>%RSD=24.4568<br>Detection Limit:0.05
Chlorogenic Acid<br>Y=(4.33994e+006)X+(-319160)<br>r^2=0.9992<br>%RSD=4.8372<br>Detection Limit:0.001	Luteolin-7-O-Glucoside<br>Y=(7.24753e+006)X+(-45563.3)<br>r^2=0.9987<br>%RSD=5.4551<br>Detection Limit:0.001
Trans-Resveratrol<br>Y=(929848)X+(-58561.0)<br>r^2=0.9994<br>%RSD=10.2395<br>Detection Limit:0.05	

Table S2. Eigenvalues of Principal Component Analysis (PCA) for volatile and phenolic components.

	F1	F2	F3	F4	F5	F6	F7
Eigenvalue	10.012	3.732	0.687	0.406	0.108	0.041	0.014
Explained Variance (%)	66.749	24.878	4.579	2.705	0.722	0.271	0.097
Cumulative Variance (%)	66.749	91.626	96.206	98.910	99.633	99.903	100.000

Table S3. Percentage contribution of volatile and phenolic variables in PCA.

	F1	F2	F3	F4	F5
Ethyl acetate	3,886	10,849	8,183	32,832	13,428
Isobutyl acetate	9,428	1,109	1,116	0,317	2,798
Ethyl isovalerate	7,976	3,953	6,895	0,231	1,070
2-Methylbutyl acetate	8,790	1,708	7,468	0,023	2,640
Ethyl decanoate	7,101	6,976	0,112	4,119	7,554
Phenethyl acetate	9,863	0,229	0,273	0,001	0,191
Acetic acid	7,286	2,504	4,604	25,245	31,593
Isoamyl alcohol	6,687	3,304	28,480	0,017	0,025
Phenylethyl alcohol	6,654	3,746	26,483	2,307	0,010
Eriotricin	8,879	0,734	1,453	15,282	3,351
Quercetin-3-O-glucoside	6,664	8,072	1,259	4,345	2,472
Quercetin-3-O-glucuronide	5,266	12,469	0,254	1,145	0,025
Quercetin-3-O-glucopyranoside	5,852	9,937	4,450	0,038	11,287
Caffeic acid	0,279	25,297	0,166	2,937	13,783
Gallic acid	5,389	9,113	8,805	11,160	9,772

Table S4. Eigenvalues of Principal Component Analysis (PCA) for volatile, phenolic, and sensory analysis.

	F1	F2	F3
Eigenvalue	19.520	4.117	1.363
Explained Variance (%)	78.079	16.470	5.451
Cumulative Variance (%)	78.079	94.549	100.000

Table S5. Percentage Contribution of volatile, phenolic and sensory variables in PCA.

	F1	F2	F3
Ethyl acetate	2,311	13,056	0,837
Isobutyl acetate	4,629	1,483	2,594
Ethyl isovalerate	4,140	3,600	3,197
2-Methylbutyl acetate	4,502	1,811	3,420
Ethyl decanoate	3,948	5,302	0,811
Phenethyl acetate	5,099	0,004	0,328
Acetic acid	5,121	0,007	0,004
Isoamyl alcohol	3,636	6,947	0,307
Phenylethyl alcohol	3,767	6,420	0,033
Eriotricin	4,862	0,345	2,704
Quercetin-3-O-glucoside	4,084	4,897	0,089
Quercetin-3-O-glucuronide	3,163	9,137	0,468
Quercetin-3-O-glucopyranoside	3,764	6,039	1,219
Caffeic acid	0,216	21,790	4,457
Gallic acid	3,135	7,518	5,765
Vinegar ID	5,004	0,407	0,474
Winy character	4,905	0,991	0,130
Fruity	4,387	1,599	5,708
Flavor intensity	4,812	1,035	1,331
Vinegar ID	4,968	0,534	0,603
Winy character	0,617	4,494	50,962
Fruity	4,952	0,331	1,452
Sourness	4,438	1,789	4,405
Pungency	4,471	0,454	7,971
Aftertaste	5,069	0,013	0,730
